# A Systematic Review Analyzing the Prevalence and Circulation of Influenza Viruses in Swine Population Worldwide

**DOI:** 10.3390/pathogens9050355

**Published:** 2020-05-08

**Authors:** Ravendra P. Chauhan, Michelle L. Gordon

**Affiliations:** School of Laboratory Medicine and Medical Sciences, College of Health Sciences, University of KwaZulu-Natal, Durban 4001, South Africa; ravendrachauhan@hotmail.com

**Keywords:** Swine influenza virus, influenza A virus, influenza B virus, influenza C virus, influenza D virus, avian-origin influenza virus, influenza pandemic

## Abstract

The global anxiety and a significant threat to public health due to the current COVID-19 pandemic reiterate the need for active surveillance for the zoonotic virus diseases of pandemic potential. Influenza virus due to its wide host range and zoonotic potential poses such a significant threat to public health. Swine serve as a “mixing vessel” for influenza virus reassortment and evolution which as a result may facilitate the emergence of new strains or subtypes of zoonotic potential. In this context, the currently available scientific data hold a high significance to unravel influenza virus epidemiology and evolution. With this objective, the current systematic review summarizes the original research articles and case reports of all the four types of influenza viruses reported in swine populations worldwide. A total of 281 articles were found eligible through screening of PubMed and Google Scholar databases and hence were included in this systematic review. The highest number of research articles (*n* = 107) were reported from Asia, followed by Americas (*n* = 97), Europe (*n* = 55), Africa (*n* = 18), and Australia (*n* = 4). The H1N1, H1N2, H3N2, and A(H1N1)pdm09 viruses were the most common influenza A virus subtypes reported in swine in most countries across the globe, however, few strains of influenza B, C, and D viruses were also reported in certain countries. Multiple reports of the avian influenza virus strains documented in the last two decades in swine in China, the United States, Canada, South Korea, Nigeria, and Egypt provided the evidence of interspecies transmission of influenza viruses from birds to swine. Inter-species transmission of equine influenza virus H3N8 from horse to swine in China expanded the genetic diversity of swine influenza viruses. Additionally, numerous reports of the double and triple-reassortant strains which emerged due to reassortments among avian, human, and swine strains within swine further increased the genetic diversity of swine influenza viruses. These findings are alarming hence active surveillance should be in place to prevent future influenza pandemics.

## 1. Introduction

Influenza viruses are the members of Orthomyxoviridae family and have a wide host range [1,2,3,4,5,6]. Due to unique physiology, swine are considered the “mixing vessel” for influenza viruses [7]. Four types of influenza viruses have been reported in swine i.e., influenza A virus (IAV), influenza B virus (IBV), influenza C virus (ICV), and influenza D virus (IDV). The genomes of IAV and IBV have eight gene segments of single-stranded negative sense RNA while the genomes of ICV and IDV have seven gene segments [8]. Among the eight gene segments of IAV and IBV, the hemagglutinin (HA) and neuraminidase (NA) are most significant and crucial for the pathogenicity of these viruses which determine the antigenic properties. The HA gene regulates the attachment of virus particles to the host receptor while NA gene regulates the release of progeny virus into the host cell. Co-infection of swine with two or more IAV strains may trigger the reassortment [9] which in turn, could facilitate the emergence of new influenza virus strains [10,11,12]. Point mutations which occur due to an error-prone RNA polymerase that lacks the ability of proof-reading and corrections during replication may also complement the genetic diversity of the influenza viruses [13]. The mechanisms of reassortment and point mutations may give rise to “antigenic shift” and “antigenic drift” within HA and NA genes, respectively, facilitating the emergence of new subtypes and lineages of influenza viruses. As a result, total 18 HA and 11 NA subtypes of IAV [14,15,16] and two lineages (Victoria/B and Yamagata/B) of IBV have been reported so far in different hosts [17,18]. 

The host range of IAV and IBV is determined by their specificity to sialic acid receptors. The HA proteins of IAV can bind to α-2,3 and α-2,6 sialic acid receptors present in avian and human trachea, respectively [19,20,21]. Interestingly, swine trachea has both, α-2,3 as well as α-2,6 sialic acid receptors, due to which swine can become infected with avian and human strains of influenza viruses [22]. 

The genomes of ICV and IDV have a gene segment termed as “hemagglutinin–esterase-fusion” (HEF) which carries out the functions similar to that of HA and NA genes of IAV and IBV. The HEF is responsible for attachment and release of ICV and IDV virus particles into the host cell [23,24,25]. The particles of both virus types ICV and IDV bind to 9-*O*-acetylated sialic acid receptors of the host [25]. Several studies have shown that human and avian origin influenza viruses can be transmitted to swine in natural settings and thus may evolve into new strains of reassorted influenza viruses [26,27].

**Figure 1:**
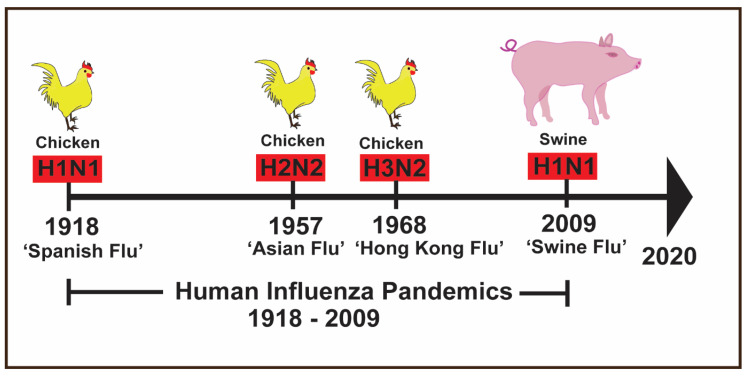
Timeline representing four human influenza pandemics between 1918 and March 2020. The first influenza pandemic known as “Spanish flu” originated in chicken in 1918. The second flu pandemic (Asian flu) and the third flu pandemic (Hong Kong flu) originated in chicken in 1957 and 1968, respectively. The most recent influenza pandemic known as “Swine flu” originated in swine in Mexico during March–May 2009.

Historically, the first flu pandemic (Spanish flu) hit the human population in 1918 [28] and killed approximately 50 million people globally [29]. The 1918 influenza pandemic emerged as a result of reassortment in which human H1 virus acquired avian (poultry) N1 neuraminidase along with internal protein genes and evolved into what is now termed as “classical H1N1” virus [30] (Figure 1). 

The second flu pandemic occurred in 1957 (Asian flu) and was traced to the H2N2 virus which killed approximately two million people [31]. The third flu pandemic hit the human population in 1968 (Hong Kong flu) with an H3N2 outbreak and killed approximately two million people [31,32]. The most recent flu pandemic (Swine flu) originated in swine in Mexico during March–May 2009 [33] and killed approximately 575,000 people worldwide [31]. The swine flu occurred due to a pandemic reassortant H1N1 virus termed as “A(H1N1)pdm09” virus [33].

Among four types of the influenza viruses, IAV is the most prevalent type and has been reported in swine in several countries. The IAV was first isolated from the nasal discharge of a swine in 1931 [34] and from the human in 1933 [35]. Strains of IAV have been reported to cause mild to severe upper respiratory tract illness in swine [36]. Strains of Victoria/B and Yamagata/B lineages of IBV were first reported in swine in the United States in 2010 [37] while the ICV in swine was first isolated in China in 1983 [38]. Both, the IBV and ICV cause mild respiratory illness in swine [39,40,41,42]. The IDV in swine was first detected in Oklahoma based swine in the United States in 2011 [5,43] which in later years has been detected in swine in China [44], Italy [45] and Luxembourg [46]. Interestingly, influenza viruses can be detected in the swine throughout the year unlike humans where seasonality affects the occurrence and progression of the disease [47,48]. 

An active surveillance of influenza viruses in swine is necessary for two basic reasons; to track the influenza virus evolution for improvements of the currently available diagnostic tests as well as for generating more effective vaccines for prevention and control of disease [49]. The currently available scientific data on swine influenza viruses would serve as a key to understand their evolutionary dynamics and transmission patterns. Therefore, this systematic review, for the first time, summarizes all four types of influenza viruses in the swine populations worldwide. 

## 2. Methods

### 2.1. Systematic Review Protocol and Search Strategy

The guidelines and the procedures as detailed by the Preferred Reporting Items for Systematic Reviews and Meta-Analysis (PRISMA) [50] were followed for drafting this systematic review. Original research articles reporting influenza virus types IAV, IBV, ICV, and IDV in swine populations until February 21, 2020 were searched through PubMed and Google Scholar databases. The influenza virus sequence information was also verified using “*Influenza Virus Resource*” of NCBI (https://www.ncbi.nlm.nih.gov/genomes/FLU/) and “*Influenza Research Database*” (https://www.fludb.org/brc/home.spg?decorator=influenza). The sequence information helped in the identification of additional relevant articles reported from Indonesia, Kazakhstan and Sri Lanka.

The search terms including “Influenza outbreak in swine” OR “Influenza A virus in swine” OR “Influenza B virus in swine” OR “Influenza C virus in swine” OR “Influenza D virus in swine” OR “Influenza virus in pigs” were entered one by one in PubMed and Google Scholar databases to identify all full-text research publications or case reports which reported influenza virus types or subtypes in swine. The outcome suggesting research publications reporting influenza virus types and subtypes in swine along with the transmission of influenza viruses between human and swine, birds and swine, poultry and swine, cattle and swine as well as horse and swine were thoroughly investigated for inclusion. The search results suggesting influenza virus prevalence and/or transmission in or between species other than swine were omitted from the analysis. Occasionally, the full-text articles were also requested from the authors, if the full-text article was not available online. Two publications which could not be accessed were omitted from the analysis. Search results yielding articles in a language other than English were omitted from the analysis.

**Figure 2:**
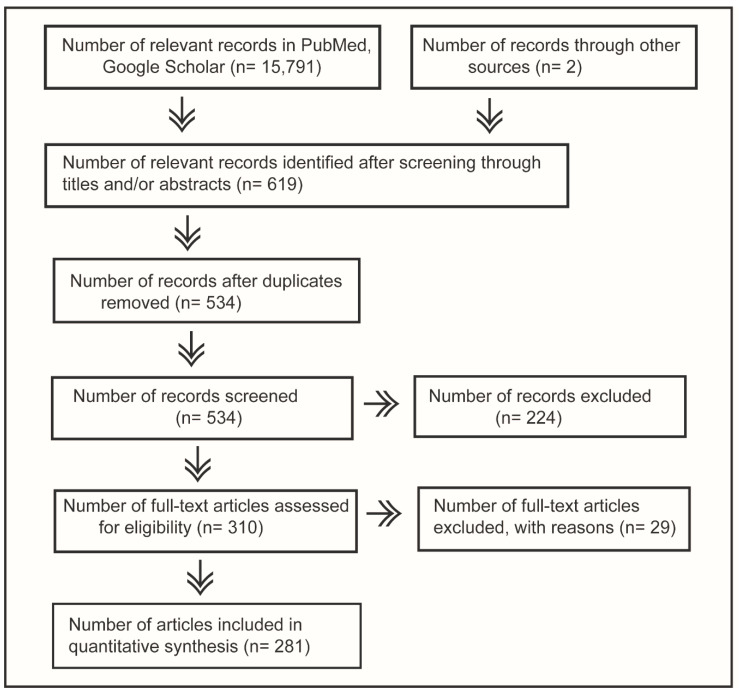
Preferred Reporting Items for Systematic Reviews and Meta-Analysis (PRISMA) chart representing the search strategy. Total 281 articles were found eligible after applying the inclusion criteria. Original research articles reporting influenza virus types influenza A virus (IAV), influenza B virus (IBV), influenza C virus (ICV), and influenza D virus (IDV) in swine populations available until February 21, 2020 were downloaded from the PubMed and Google Scholar databases.

The articles were first screened through their abstracts to find out their relevance for inclusion, and, if required, the introduction and/or results and discussion sections were also screened to assess their relevance for inclusion. The relevant articles were downloaded and stored on the computer drive for further screening and refinement according to PRISMA guidelines [50]. The references of downloaded publications were also screened to identify relevant articles reporting the influenza viruses in swine, which were also downloaded to include in the analysis. An overview of the methodology can be observed in the PRISMA chart (Figure 2). 

### 2.2. Inclusion and Exclusion Criteria

The following criteria were applied for screening of the eligible articles:

The original research articles and case reports which documented the influenza viruses in swine in natural settings across the world were included in the analysis. 

The experimental studies which did not report the natural cases were excluded from the analysis.

The reviews, letters, editorials, conference proceedings, and articles in a language other than English were not included in the analysis. Duplicate articles were also excluded from the analysis.

The eligible articles (*n* = 281) thus selected were included in the analysis for this systematic review.

### 2.3. Ethical Approvals

This systematic review did not involve animal sampling or experimental protocols in the laboratory. The data used for writing this article were obtained from the PubMed and Google Scholar databases. This systematic review is part of a research project which has already obtained the relevant ethical approvals from the Animal Research Ethics Committee (AREC), University of KwaZulu-Natal, Durban, South Africa; AREC Reference: AREC/041/019D. Additionally, the authors have the required permission to do research in terms of Section 20 of the Animal Diseases Act, 1984 (Act No. 35 of 1984) from the Department of Agriculture, Forestry and Fisheries (DAFF), Government of the Republic of South Africa; DAFF Reference: 12/11/1/5/4 (1425). 

## 3. Results

**Figure 3:**
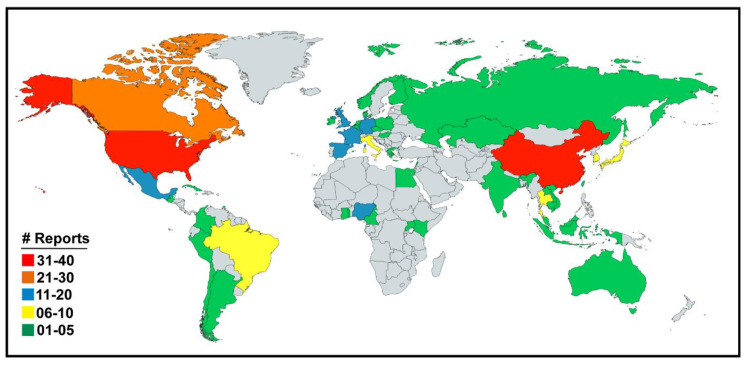
The world map represents the prevalence of influenza viruses i.e., IAV, IBV, ICV, and IDV in swine populations until February 2020. Highest number of articles were reported from the United States (*n* = 40), followed by China (*n* = 39), Canada (*n* = 24) and other countries. The world map was created online at https://mapchart.net.

The original research articles and case reports on the serological and virological prevalence of all the four genera of influenza viruses i.e., IAV, IBV, ICV and IDV were downloaded, analyzed and summarized in the region-specific manner across the world. Influenza viruses have been reported from 53 countries located across six continents (Figure 3; Table 1) until February 2020. 

### 3.1. Influenza Viruses in Swine in Africa 

#### 3.1.1. Cameroon

The first report of IAV in Cameroonian swine appeared when A(H1N1)pdm09 virus was documented during 2009–2010. The youngest infected swine was four-month old which suggested that A(H1N1)pdm09 virus can infect the young piglets [51]. Nine more swine herds in Cameroon were found infected with A(H1N1)pdm09 and H1N1 viruses during May–June 2011 [52]. A multiple-site study including free-roaming and penned swine along with domestic poultry and Columbiformes birds between December 2009 and August 2012 identified one IAV positive swine at each of the two study sites. The inter-species transmission of IAV was ruled out as all the birds were negative for the IAV [53]. 

#### 3.1.2. Nigeria

The first evidence of the past IAV infection in Nigerian swine appeared in 2008 when H1N1 and H3N2 virus antibodies were detected in swine sampled at three different locations [54]. Shortly after that, in 2009, the first report of A(H1N1)pdm09 virus appeared in the Nigerian swine when one swine herd was found seropositive for A(H1N1)pdm09 virus. Interestingly, eight other swine herds were found seropositive for the H1N1 and four herds were found positive for human-like H3N2 viruses. The seroprevalence of IAV further increased as 66 swine herds were detected positive for A(H1N1)pdm09 virus and 53 herds were found seropositive for H1N1 virus in 2012 [52]. The active infection (viral RNA) of A(H1N1)pdm09 virus was first reported in the Nigerian swine between July 2010–June 2012 when 18 A(H1N1)pdm09 virus isolates were retrieved from the swine in Lagos. The zoonotic transmission of A(H1N1)pdm09 virus to the exposed human workers was ruled out as all the human samples were negative for the A(H1N1)pdm09 virus [55]. 

Nineteen more A(H1N1)pdm09 and five human-origin H3N2 viruses were identified in the Nigerian swine during 2013-2015 [56]. Later one more report of the human strain of H3N2 virus appeared in swine during January–February 2014 [57]. A high seroprevalence of IAV in a commercial piggery was reported in Lagos. Total 197 human and 281 swine sera samples were screened which determined that 87% human and 67% swine sera had antibodies for IAV depicting the past exposure [58] but the active infection was absent given that all nasal swabs were negative for IAV infection [58]. Lately, highly pathogenic avian influenza virus (HPAIV) strain H5N1 was detected in 22 swine samples between December 2015 and February 2016 during an ongoing H5N1 disease outbreak in Nigerian poultry [59] which indicated the inter-species transmission of H5N1 virus from poultry to swine [59]. 

#### 3.1.3. Egypt

A molecular study reported the negative prevalence of avian-like H5N1 and H5N2 viruses in Egyptian swine in May 2008 [60] but the serological investigation identified H5N1 virus antibodies in seven and H5N2 virus antibodies in four swine sera samples [60] which suggested a past exposure of these swine to the viruses. The active H5N1 infection in Egyptian swine was again ruled out in October 2013 as the viral RNA could not be detected in 36 swine samples but interestingly, the antibodies against avian-like H5N1, H9N2, and A(H1N1)pdm09 viruses were detected in swine sera samples which suggested a past exposure [61]. Interestingly, 122 of the 157 swine nasal swab samples collected during 2014 and 2015 were found positive for IAV active infection using RT-PCR. As a result, HA subtyping identified 46 avian-origin H5N1, seven H9N2 and 69 A(H1N1)pdm09 viruses [61].

#### 3.1.4. Kenya

The first report of IAV in swine in Kenya appeared in May 2010 when A(H1N1)pdm09 virus were detected in eight swine samples collected from the Asembo and Kibera counties and at a Nairobi based abattoir. The extended serological study further identified H1N1 and H3N2 viruses in swine during August 2011 to December 2012 [62]. The active IAV infection was reported in four household members having acute respiratory illness while the backyard swine were negative for the IAV and IBV in Kiambu county during September 2013–August 2014. On the contrary, the serology identified the IAV antibodies in 230 swine sera samples suggesting a past exposure of IAV [63]. The A(H1N1)pdm09 virus was again reported in five swine samples collected from a slaughterhouse in Kenya during September 2013–September 2014. Interestingly, all the 288 human subjects including the slaughterhouse workers or the traders and farmers who had visited the slaughterhouse were negative for IAV, hence ruled out the zoonotic transmission [64]. 

#### 3.1.5. Other African Countries

Swine in Benin and Cote d’Ivoire reported no prevalence of IAV during 2009–2010 [65] while A(H1N1)pdm09 virus was detected in swine in Reunion Island during 2009–2011 [66] and in Togo during October 2012–January 2014 [67]. The human strain of H3N2 virus was detected in swine in Ghana during January–February 2014 [57]. One more report documented IAV in swine in two districts of Uganda in 2015 [68]. 

**Figure 4:**
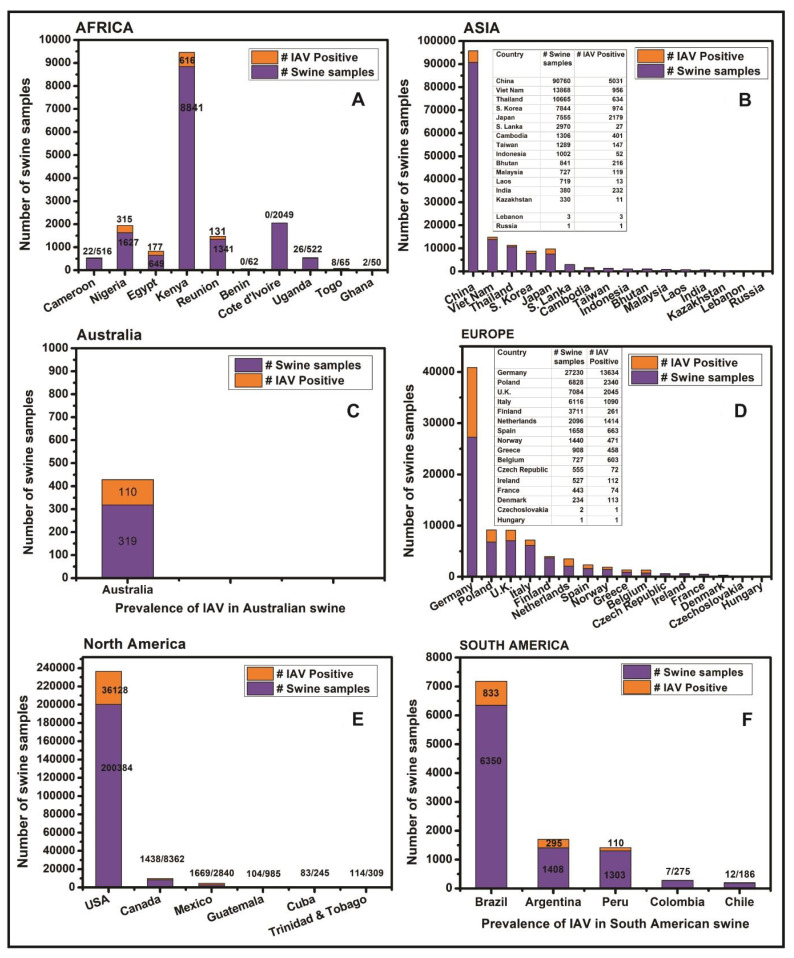
Country-specific prevalence of IAV in swine populations worldwide. (**A**) IAV prevalence in African swine population with highest prevalence reported from Kenya. (**B**) IAV prevalence in Asian swine where most cases were reported from China. (**C**) IAV prevalence in Australian swine. (**D**) IAV prevalence in European swine population. Germany reported the highest number of cases in Europe. (**E**) IAV prevalence in North American swine with most of the cases in the United States. (**F**) IAV prevalence in South American swine with most cases reported from Brazil. The variations in the prevalence of influenza viruses in swine populations among countries may be because of certain factors including (a) swine populations differ greatly among countries; (b) surveillance efforts differ greatly among countries; (c) the non-English publications were excluded from the analysis. These graphs do not represent the severity of disease as the swine populations and the objectives of surveillances may vary among countries.

Overall, influenza viruses have been reported in swine from eight African countries including Cameroon, Nigeria, Egypt, Kenya, Reunion island, Togo, Ghana, and Uganda (Figure 4A). The A(H1N1)pdm09 virus, which originated in Mexican swine in 2009, has been reported in all except Ghana and Uganda. Interestingly, the HPAIV strain of H5N1 has been reported in swine in Nigeria and Egypt while HPAIV strain H5N2 and low pathogenic avian influenza virus (LPAIV) strain H9N2 have also been reported in the Egyptian swine (Table 1). 

### 3.2. Influenza Viruses in Swine in Asia

#### 3.2.1. China

China is considered the epicenter of influenza viruses [69]. The first seroprevalence of IAV in Chinese swine was documented during 1977-1982 when antibodies for 38 H1N1, 22 H3N2, 12 H4N6, 12 H5N3, and seven H9N2 viruses was detected in swine sera obtained from apparently healthy swine [70]. The first ever report of ICV in swine was documented from the apparently healthy swine in Beijing when 15 ICV isolates were retrieved during January–December 1981 [38]. Three isolates of reassortant H1N2 virus were identified after an influenza-like illness triggered abortions and mortalities in sows on a swine farm in November 2004 [71]. The same year, LPAIV strain H9N2 was isolated from the sick or dead swine in China which was the first ever isolate of H9N2 virus retrieved from a swine [72]. 

First human-origin H1N1 and four human-origin H3N2 virus isolates in Chinese swine were retrieved during 2005–2006 [73]. Further, two isolates of swine H3N2 viruses, four isolates of avian-origin HPAIV strain H5N1 and two isolates of H1N1 viruses were detected in swine nasal swab and lung tissue samples collected from swine in central provinces of China during 2004–2006 [74]. Surprisingly, two isolates of equine influenza virus H3N8 were also detected in swine during December 2005 and January 2006 [74]. Another report of avian-origin H9N2 virus in Chinese swine was documented during 2006–2007 when four H9N2 virus isolates with closely related nucleotide sequences were retrieved from swine [75]. Each of the two different investigations reported 19 H1N1, one H1N2 and nine H3N2 virus isolates from Chinese swine during 2006–2009 [76,77]; the H1N2 virus and all nine isolates of H3N2 viruses were either double or triple-reassortant viruses [76]. 

The first report of HPAIV strain H5N1 in swine was documented during October 2008–May 2009 when two H5N1 virus isolates were retrieved from apparently healthy swine [78]. The third report of avian-origin H9N2 virus in Chinese swine appeared when 144 apparently healthy swine across four provinces viz., Yunnan, Guangdong, Fujian and Zhejiang were found H9N2 positive over a four-year period during March 2008–March 2012. The frequent interactions of birds to the swine at the study sites was reported which was suspected to be the most likely source of infection [79]. Further, a novel strain of avian-origin H4N1 virus was isolated from a Chinese swine in 2009 [80].

Several classical and avian-like H1N1, Eurasian avian-like H1N1, triple-reassortant H1N1, H1N2, H3N2 and A(H1N1)pdm09 viruses were reported in Chinese swine between 2009 and 2016 [81,82,83,84,85,86,87,88,89,90,91]. A triple-reassortant H1N1 virus having the internal genes of avian, human, and swine lineages of influenza viruses was reported from a two-month old piglet on a Guangdong based swine farm in January 2010 [92]. Three reassortant H3N2 virus isolates having internal genes of A(H1N1)pdm09 virus were reported in swine between November 2010 and June 2011 [93].

A three-year old boy was diagnosed with European origin avian-like H1N1 virus on a family swine farm in a rural area of the Jiangsu province in December 2010 which speculated a zoonotic transmission from swine to the boy [94]. The first report of H10N5 avian-origin influenza virus in a domestic swine in Hubei province further extended the diversity of swine influenza viruses and provided another evidence of interspecies transmission of avian influenza virus to the swine under natural conditions [95]. Several other avian-origin H3N2, H4N8, H6N6, H7N9, H5N1, and H9N2 virus antibodies were detected in swine in China during April 2010–June 2014 [86,96,97,98]. 

Another interspecies transmission of avian-like H1N1 virus in southern China was observed when 219 swine and 61 swine farm workers were identified to be infected with avian-like H1N1 swine influenza virus between March 2011 and March 2013 [99]. Further a zoonotic transmission of H9N2 virus was identified at a Shandong based swine farm during May 2013–April 2014 when H9N2 virus antibodies were detected in 84 swine and four farm workers. The wild birds visiting swine feeding sites at the swine farm were speculated to serve as the carrier for H9N2 virus [100]. Zoonotic transmission of H1N1 virus was reported on a swine farm in Shandong province between March 2015 and February 2016 among the swine exposed human workers having influenza-like illness. As a result, five of the 32 (15.6%) nasal swab samples were found IAV positive; a married couple exposed to swine were found infected with H1N1 virus [88].

The IAV infection was also documented in 44 wild boars in Jilin province of China between April 2015 and February 2016 [101]. The first report of the IDV prevalence in Chinese swine documented 21 IDV positive swine in the Guangdong province in 2016 [44]. The swine IDV sequences shared high similarity (99–100%) with IDV sequences reported earlier from the bovine species in China [102] which indicated the transmission of IDV from bovine to swine in China.

#### 3.2.2. Hong Kong and Tibet

Hong Kong is a special administrative region while Tibet is an autonomous administrative region under the control of People’s Republic of China. The H1N1 and H3N2 virus isolates were successfully retrieved from apparently healthy swine in Hong Kong during July 1993–June 1994 [103]. Further, classical swine H1N1, H3N2 and avian-like H9N2 viruses were identified in Hong Kong based swine between March 1998–June 2000; two independent introductions of the avian-like H9N2 viruses were ascertained from avian species to the swine [27,104,105]. 

The first information of IAV seroprevalence in Tibetan swine appeared during April–December 2010 when antibodies against H1N1 and H3N2 viruses were detected in swine sera collected from Tibet [106]. 

#### 3.2.3. Bhutan

The first report of H1N1 seroprevalence in swine in Bhutan appeared when H1N1 virus was detected in backyard as well as breeding swine during October 2011 and February 2012 [107]. 

#### 3.2.4. Cambodia

The H1N1 virus was reported in swine over a five year-period between 2006–2010 while the A(H1N1)pdm09 and H3N2 viruses were identified only in 2010 [108]. Later three triple-assortant H3N2 viruses were isolated and sequenced from the backyard swine between May 2011 and July 2012 [109]. 

#### 3.2.5. Japan

The antibodies against A/Hong Kong(H3N2) virus termed as “*A/Swine/Wadayama/5/69*” were first detected in Japanese swine in 1969 [110,111]. The H3N2 virus seroprevalence in Japanese swine was further documented in Sendai City during 1977 to 1980 [112]; the transmission between human and swine was also suggested [112]. The first active IAV infection was reported when two reassortant H1N2 virus isolates were retrieved from the Japanese swine having influenza-like disease in 1978. The isolated H1N2 virus was believed to be a recombinant of H1N1 and H3N2 viruses [113]. Further 340 swine were diagnosed with H1N1 antibodies in Toyama Prefecture between 1978–1982. A lower seroprevalence was observed during the summer months while the seroprevalence was relatively higher during the winter season [114]. Again, one more H1N2 virus was isolated and characterized from the sows in Ehime Prefecture in September 1980 [115]. Intriguingly, 18 H1N1, H1N2 and H3N2 viruses were detected in swine imported from the United States, however, all the imported swine from the Europe were negative for the IAV infection. This was the first report of the IAV infection in the imported swine [116]. 

The ICV seroprevalence (19%) in Japanese swine was first reported in the Hyogo Prefecture during July 1981–June 1982 [117] but swine in Yamagata Prefecture were found seronegative for the ICV between August 1979 and March 1986 which suggested a localized transmission of ICV in swine within Hyogo Prefecture [118]. 

Several other reassortant H1N2 virus isolates were reported in Japanese swine after 1991 [119]. One novel reassortant H1N2 virus appeared to have emerged from the A(H1N1)pdm09 virus was reported in swine in Gunma Prefecture while two other H1N2 viruses appeared to have emerged from the Japanese H1N2 viruses with internal genes from A(H1N1)pdm09 virus. One more H1N2 virus was detected in swine which was closely related to the Japanese H1N2 virus [120]. 

The immunohistochemistry identified lesions in the lungs of the sick swine infected with reassortant H1N2 virus [121]. Additionally, several H1N1 and H3N2 viruses have also been reported in Japanese swine between 1990 and 2017 [122,123]. Interestingly, six H1N1 virus isolates were identified with reassorted genes from A(H1N1)pdm09 virus while one H1N1 isolate appeared to have H1 gene from Japanese swine influenza virus with internal genes of A(H1N1)pdm09 virus. Further, one H3N2 virus isolate was determined to have genes of Japanese swine influenza and A(H1N1)pdm09 viruses [124]. These results reflected the occurrence of the reassortment events between Japanese swine influenza and A(H1N1)pdm09 viruses. 

IAV seroprevalence has lately been reported in wild boars (*Sus scrofa leucomystax*) in Japan. Three wild boars in the Yamaguchi Prefecture were found seropositive for A(H1N1)pdm09 virus while nine wild boars in Tochigi Prefecture were seropositive for the swine H1N1 virus. But, the active IAV infection could not be identified in these wild boars as all the nasal swab samples were negative for IAV and IBV [125]. In a more recent investigation, fifteen wild boars were found seropositive for A(H1N1)pdm09 virus in Kagoshima Prefecture between November 2014–December 2017 while two of these fifteen wild boars had antibodies against H1N2 and H3N2 viruses as well [126]. This reflected a past exposure of the Japanese wild boars to the IAV strains. 

#### 3.2.6. South Korea

The first active IAV infection in the Korean swine was identified in December 1998 when three H3N2 virus isolates were recovered from the swine experiencing an acute influenza-like respiratory disease. The close relatedness of these Korean swine H3N2 isolates with human-origin H3N2 viruses reported from Korea between 1987–1999 suggested the events of reverse zoonosis [127]. One unique H7N2 virus isolate was detected in swine which had seven gene segments originated from Hong Kong avian-origin H7N2 virus isolated in 1978 and the NS gene originated from Hong Kong H5N3 virus isolated in 1977. Additionally, four typical swine influenza H1N1 viruses were identified in swine [128]. 

Several H1N1, H1N2, and H3N2 viruses were detected in symptomatic South Korean swine after 2000 [129,130,131,132,133,134]. The IAV localization in the swine lung tissues was confirmed by immunohistochemistry [130]. Total 35 avian-origin H5N2 viruses of Eurasian lineage were identified in swine in different South Korean provinces during 2004–2008 which suggested cross-species transmission of H5N2 virus [135]. 

Three H1N1 virus isolates closely related to US isolates of H1N1 were obtained from 45-day-old piglets in Korea in January 2005. The other swine farms in the proximity of this index farm were negative for the H1N1 virus [136]. Further, one H1N1, two H1N2, and one H3N2 subtypes of IAV identical to the American strains based on their HA and NA gene sequences were obtained from swine nasal swab, lung, and thoracic fluid samples during 2005–2006 which suggested that there was no probability of arising of these IAV strains in Korea through recombination [137]. 

Two novel isolates of swine H3N1 virus with high genomic similarity to each other were retrieved from two different swine farms in Korea during March–April 2006 which would be due to a common origin of these isolates. These viruses had human-like H3 gene while other gene segments originated from swine influenza viruses within Korea. High reactivity of the 52 swine sera samples to H3N1 virus antibodies suggested a previous exposure and probability of the swine to swine transmission of H3N1 virus [138]. 

The human to swine transmission of A(H1N1)pdm09 virus was reported in Chungbuk province where 42 A(H1N1)pdm09 virus isolates were recovered from swine lung tissues [139]. The reassortment between A(H1N1)pdm09 and swine H1N2 viruses emerged into a novel reassortant H1N2 virus in swine [140]. A triple-reassortant H3N2 virus was identified in swine during December 2011–May 2012 which indicated the IAV reassortment was taking place in Korean swine [141]. A swine fever eradication campaign identified nine A(H1N1)pdm09, two classical H1N1 and one H1N2 viruses in wild boars which were hunted and killed in South Korea during 2012 [142]. More recently, a complete genome sequence of H1N1 virus was reported from a domestic swine in Korea in 2016 [143].

#### 3.2.7. Thailand

The occurrence of IAV in Thai swine was first reported during November–December 1978. Active H3N2 infection was detected in one swine while several other swine had H3N2 antibodies [144]. Two H1N1 virus isolates from Thai swine were first recovered in January 1988 [145]. Several studies reported H1N1, A(H1N1)pdm09, H1N2, and H3N2 viruses in swine exhibiting respiratory disease symptoms between 2000 to 2014. Intriguingly, one swine sample was found co-infected with four IAV subtypes including H1N1, H1N2, H3N1, and H3N2 viruses [146,147,148,149,150,151,152]. 

The first evidence of H5N1 seroprevalence in Thai swine was documented in 2004 when eight H5N1 positive swine sera samples were identified [153]. Later ten H1N1 and two H3N2 virus isolates were retrieved from piglets aged between 4 to 12 weeks during 2008–2009 [154]. Interestingly, most of the virus isolates retrieved in this study were obtained from 4 to 8 week-old piglets which was in agreement of a previous report stating that swine influenza viruses can be successfully retrieved from piglets less than ten weeks of age [155]. 

A zoonotic transmission of IAV was reported at a Thai swine farm where all the swine were found positive for either H1N1 or H1N2 virus. Interestingly, two farm owners, 46 swine handlers, four veterinarians, five farm cleaners and two farm office workers also reported IAV seroprevalence. This study claimed that there was transmission of swine influenza viruses from swine to human however the possibility of human to swine transmission was ruled out [156]. 

After a respiratory disease outbreak in nursery piglets, 15 nasal swabs were found positive for A(H1N1)pdm09 virus between December 2009 and March 2010. Fifteen sera samples of the farm workers along with three sera from dogs and one serum obtained from a cat were negative for IAV, hence the interspecies transmission of IAV was ruled out [157]. 

The first report of active infection with reassortant H1N1 virus in Thai swine appeared in February 2010 but the follow up screenings conducted after two and three months, respectively confirmed the cessation of the active infection as the viral RNA was not detected anymore [158]. 

The reshuffling and reassortment of IAV internal genes were reported in Thai swine in February 2012. The HA and NA genes of H1N1 virus isolates clustered with the Eurasian swine-like IAV lineage while the H3N2 viruses diverged and formed a separate group. All the internal genes of H1N1 and H3N2 virus isolates appeared to be derived from A(H1N1)pdm09 viruses which confirmed the events of reassortments [159].

#### 3.2.8. Vietnam

The events of reverse zoonoses were suggested after the detection of A(H1N1)pdm09 virus seroprevalence in Vietnamese swine during October 2009–March 2010 [160]. One more evidence of reverse zoonosis was identified during February–March 2010 after six triple-reassortant H3N2 viruses having a novel cluster of the Triple Reassortant Internal Gene (TRIG) cassette were isolated. The HA and NA genes of these reassortant H3N2 isolates originated from human H3N2 viruses reported between 2004–2006 while the other six internal genes had a high similarity with the Korean and American isolates [161]. 

Two more studies reported the H1N1, A(H1N1)pdm09, HIN2, and H3N2 virus isolates during February 2010–December 2013 from clinically healthy swine with no influenza disease symptoms [162,163]. Additionally, the antibodies for A(H1N1)pdm09 and H3N2 viruses were detected in swine which suggested a past exposure of swine to these viruses [163]. 

#### 3.2.9. India

A high seroprevalence of H1N1, H2N2 and H3N2 viruses was detected in human and swine sera in Calcutta, India during 1982–1990 [164]. The first active infection of IAV in Indian swine appeared in 2009 when A(H1N1)pdm09 virus isolates were reported from a swine farm located in Uttar Pradesh. Interestingly, the retrieved A(H1N1)pdm09 virus sequences were similar to the North American and Korean viruses which might be either because of trade or long-distance transmission [165]. 

#### 3.2.10. Lebanon

After an influenza outbreak on Lebanese poultry farms in 2005 the farmers fed the carcasses of the dead flocks to the swine. Intriguingly, a following investigation found that three swine were seropositive for the H9N2 virus while approximately one-third of the poultry farm workers were seropositive either for H1 or H9 viruses [166]. These results revealed the interspecies transmission of IAV among poultry, farm workers and swine.

#### 3.2.11. Malaysia

The seroprevalence of H1N1 and H3N2 viruses in four to six-month-old Malaysian swine at 41 swine farms was reported during May–August 2005. Co-infections of H1N1 and H3N2 were detected in 29 swine samples [167]. 

#### 3.2.12. Laos

The seroprevalence of H3N2 virus in swine samples obtained from the slaughterhouses in Laos was reported between May 2008 to January 2009 [168]. 

#### 3.2.13. Russia

A full-length genome sequence of a reassortant H1N1 virus was reported from a Russian swine in 2016. The HA and NA genes of this virus isolate shared 90% identity with the H1N1 viruses that were reported from humans in the USA in the 1980s [169]. 

#### 3.2.14. Taiwan

The human to swine transmission of IAV was speculated after IAV antibodies were detected in 147 Taiwanese swine during June 1969–May 1970. The results were further confirmed with virus isolation which retrieved 13 IAV isolates [170]. More recently, IBV of Victoria/B lineage was detected in swine nasal swab samples collected from apparently healthy swine at three swine farms in 2014 [171]. 

#### 3.2.15. Indonesia

An active IAV infection in 52 swine within four provinces in Indonesia was identified during 2005–2009. Interestingly, 39 H5N1 virus isolates were successfully retrieved and sequenced [172]. 

#### 3.2.16. Sri Lanka

The first report of influenza in Sri Lankan swine was documented during 2004–2005 after one human-like H3N2 virus was identified. Later, A(H1N1)pdm09 virus isolates were identified in swine during 2009–2012. A spillover of these viruses from human to swine was speculated [173]. 

#### 3.2.17. Kazakhstan

One recent investigation in Kazakhstan during 2017–2018 identified nine H1N1 and eight H3N2 viruses in human while seven H1N1 and four H3N2 viruses were identified in swine. Interestingly, 10 of the human samples were also positive for IBV infection while the swine samples were negative for IBV [174]. 

In summary, the influenza viruses have been reported in swine in 16 Asian countries including China, Japan, Thailand, South Korea, Viet Nam, Cambodia, Taiwan, India, Bhutan, Russia, Laos, Malaysia, Lebanon, Indonesia, Kazakhstan, and Sri Lanka (Figure 4B). Apart from the most common IAV strains of H1N1, H1N2, H3N2, and A(H1N1)pdm09 viruses, several avian-origin H5N1, H5N3, H4N1, H4N6, H4N8, H6N6, H7N9, H9N2, and H10N5 influenza viruses were also reported in Chinese swine. Horse to swine transmission of equine influenza virus H3N8 was reported in China. Additionally, avian-origin H7N2, H5N2 viruses were identified in South Korean swine while H5N1 was reported in Indonesian swine. Interestingly, after the swine were fed upon dead poultry carcasses in Lebanon the H9N2 virus was detected in Lebanese swine. The IBV was reported in Asian swine only in Taiwan while strains of ICV were reported in swine in China and Japan while IDV was recently reported in Chinese swine (Table 1).

### 3.3. Influenza Viruses in Swine in Australia

Swine influenza was first reported in Australian swine only in 2009 after a swine farm owner reported coughing symptoms among swine. Simultaneously, some of the human workers on the farm also developed influenza like symptoms and hence stayed out of the farm until recovery. Later, the farm owner also developed similar symptoms following which he was tested for A(H1N1)pdm09 virus which resulted positive. As a result, a representative number of swine showing coughing symptoms and loss in appetite were sampled for molecular diagnostics and serology which confirmed that 12 swine were positive for H1N1 virus [175]. 

Second report of IAV in Australian swine appeared on a Queensland farm in August 2009 when a veterinarian observed elevated temperature, coughing and loss of appetite in swine. Simultaneously, two of the staff members on the farm exhibited influenza-like symptoms and hence were sampled for diagnostic testing using nasal swabs. Interestingly, both the staff members and four of the swine were found positive for the A(H1N1)pdm09 virus. Sequencing identified that the HA gene of A(H1N1)pdm09 virus retrieved from a staff member was identical to the virus retrieved from the swine which suggested transmission of A(H1N1)pdm09 virus between swine and human [176]. 

Third report of IAV in Australian swine appeared when a respiratory disease outbreak in swine and the farm workers occurred in Perth, Western Australia during 2012 which identified 43 IAV positive swine. Sanger sequencing of HA and NA genes identified six novel HIN2, three novel H3N2, one A(H1N1)pdm09 and two seasonal H3N2 viruses in swine. On the contrary, only one out of eight human workers were found positive for seasonal H3N2 virus. This study could not conclude the event of zoonotic transmission of IAV between swine and human workers at the farm [177]. 

The fourth report of IAV was documented when 14 IAV positive swine were identified at a commercial swine farm in Western Australia during July–September 2012 and later during September–November 2016. Additionally, 17 swine were determined to be IAV positive in southern Queensland. The complete genomes of 10 IAV isolates retrieved in Western Australia and Queensland were successfully sequenced which identified seven H1N2, two human-like H3N2 and one H1N1 virus [178]. 

Overall, four reports of IAV outbreaks in swine in New South Wales, Queensland and Western Australia were available (Figure 4C). The H1N1, H1N2, H3N2 and A(H1N1)pdm09 subtypes have been reported from Australian swine with relatively low prevalence. 

### 3.4. Influenza Viruses in Swine in Europe

#### 3.4.1. Belgium

The H1N1 virus was identified in swine lung tissues or trachea of two of the deceased sows after an influenza-like disease erupted at two swine farms in January 1979. Interestingly, it was also reported that the identical virus was detected in wild ducks in Germany [179]. Since it was already established that H1N1 from wild ducks can successfully infect swine if inoculated via intranasal route [179] hence this observation suggested the transmission of H1N1 from wild ducks to the swine [180]. A second investigation isolated three avian-like H1N1, two H1N2 and twelve human-like H3N2 viruses from eight commercial swine farms in March 1999 [181]. 

#### 3.4.2. Denmark

Denmark has been running a passive surveillance program for IAV detection in swine since 2011. The H1N2 virus having the H1 gene which evolved from H1N1 avian-like viruses and N2 gene which evolved from human H3N2 viruses was reported in swine during 2011–2013 [182]. This was an example of how IAV can evolve through reassortment and may emerge into a new IAV strain. 

The other investigation included swine sampling at different time intervals to assess the persistence of IAV shedding in Danish swine which detected one avian-like H1N1 and 107 reassortant H1N2 viruses. This study observed that most of the swine were shedding IAV right before achieving six weeks of age. Surprisingly, a piglet as young as just three days was found infected with IAV [183]. 

Two H3N2 isolates having H3 genes from seasonal human influenza along with internal genes that originated from A(H1N1)pdm09 virus and NA genes from contemporary N2 swine influenza viruses that have been in circulation in Denmark were retrieved from young piglets at two locations during 2011–2014 [184]. H3N2 virus was also detected from piglets having respiratory illness and from sows with reproductive problems in commercial piggeries in 2014 [184].

#### 3.4.3. United Kingdom

The H3N2 virus antibodies were first detected in English swine in 1973 which revealed the past exposure of swine to H3N2 virus [185]. Later, the antibodies for H1N1 and H3N2 viruses were detected in swine at a slaughterhouse in England during 1991–1992 [186]. Interestingly, this serological investigation also reported the antibodies for IBV in eight and for ICV in 198 swine [186]. 

A molecular investigation identified a novel H1N7 virus in swine in England which had six of its RNA segments closely related to those of human viruses while two RNA segments were identical to those of equine viruses which concluded that the H1N7 strain may have evolved due to reassortment between human H1 and equine H7N7 viruses [187,188]. 

The first report of A(H1N1)pdm09 virus in English swine appeared in September 2009 when histology and immunofluorescence assays followed by molecular diagnostics and sequencing confirmed four A(H1N1)pdm09 virus infected swine in the Northern Ireland [189]. After this, 17 more A(H1N1)pdm09 virus isolates were reported in swine in England during September 2009–October 2010 which revealed that A(H1N1)pdm09 virus was in circulation in English swine during the 2009 flu pandemic [190]. The same year, four H1N2 virus isolates were reported in English swine which had six internal genes of A(H1N1)pdm09 virus along with HA and NA genes of H1N2 virus hence were identified as the novel reassortant H1N2 strains [191]. In a more recent study, two more IAV positive swine were identified in the United Kingdom in 2016 [192]. 

#### 3.4.4. Finland

However the first report of seroprevalence of H1N1 virus in Finnish swine appeared in 2008 during an investigation which detected H1N1 virus antibodies in swine at seven swine farms which further increased to 24 swine farms in 2009 [193] but the first isolate of avian-like swine H1N1 virus (indicative of active infection) was detected from the lung tissues of a swine in February 2009. Later, the first A(H1N1)pdm09 virus in Finnish swine was detected in November 2009 [193]. Three more swine were identified with IAV antibodies during May 2011–January 2014 which was due to a past exposure to IAV [194].

#### 3.4.5. France

The H1N1 viruses in turkey and swine were identified after the swine influenza outbreak hit the turkey population in Brittany, France in February 1983 which suggested that IAV transmission happened from swine to turkey [195]. Later two strains of H1N2 virus were isolated from six swine exhibiting influenza-like illness in Brittany during 1987–1988 [196]. Another investigation reported H1N1, H1N2, and H3N2 viruses in swine herds experiencing respiratory disease in Brittany region [197].

A negative prevalence of IAV was reported in wild boars in Camargue during September 2009–November 2010 given that all the 315 nasal swabs obtained from either hunted or trapped wild boars along with all the sera samples were negative for IAV [198]. 

A more recent investigation reported the zoonotic transmission of A(H1N1)pdm09 virus from swine to a farmer in January 2018. This farmer along with a veterinarian collected nasal swab samples from three pregnant sows exhibiting influenza-like illness on the swine farm and submitted to a local diagnostic laboratory for analysis which, as a result, were found IAV positive. Few days later, the farmer and the veterinarian both developed the influenza-like symptoms. The farmer was later diagnosed with A(H1N1)pdm09 virus [199]. 

#### 3.4.6. Germany

Sixty-five IAV positive wild boars were identified across five German states during 1997–2006. Cloning and sequencing identified H1N1 and H3N2 viruses in these wild boars [200]. Later thirteen H1N1, three reassorted A(H1N1)pdm09 and four H1N2 viruses were detected in swine during 2009–2010. Interestingly, the A(H1N1)pdm09 virus isolates had high similarity with the A(H1N1)pdm09 viruses reported earlier in humans within Germany which suggested a reverse zoonotic transmission of the A(H1N1)pdm09 virus [201]. 

A nationwide sero-surveillance identified 12,585 swine with H1N1, 9,566 swine with human-like H1N2, 12,220 swine with human-like H3N2 and 11,086 swine with A(H1N1)pdm09 virus antibodies during June 2009–December 2012 which reflected a high seroprevalence of influenza viruses in German swine population [202]. 

Later 273 IAV positive swine exhibiting influenza-like illness were detected between January 2010–December 2013. Subtyping successfully distinguished 198 of 273 samples into H1N1, H1N2, H3N2 and A(H1N1)pdm09 viruses. The H1N1 virus was the most widely occurring in German swine while A(H1N1)pdm09 virus had the lowest prevalence [203]. 

#### 3.4.7. Greece

The H1N1, H1N2, H3N2, and A(H1N1)pdm09 viruses were detected in swine sera samples collected from apparently healthy swine at 42 swine farms during 2002–2004 and from 46 swine farms during 2010–2012 [204]. 

#### 3.4.8. Italy

The seropositivity of Italian swine to H3N2 virus was first reported during December 1976–November 1977 when 24 swine were detected with H3N2 antibodies [205]. The first report of H1N1 active infection in Italian swine appeared during an influenza disease outbreak between 1977 to 1986 which identified 63 H1N1 viruses [206]. Further, four H3N2 viruses were detected in swine nasal swabs originated from three swine farms and an abattoir during 1981–1982 [207]. 

Later 47 H1N1 and 37 H3N2 viruses were detected in swine during 1992–1995. Interestingly, four human sera samples were also positive for H1N1 and 77 samples were positive for H3N2 viruses which might be due to the transmission between human and swine [208]. Further IAV seroprevalence was detected in the age group of three-month to four-year old swine during 2002–2004 [209]. 

The first report of A(H1N1)pdm09 virus in Italian swine appeared after a respiratory disease outbreak in piggeries in Lombardia region of Northern Italy in November 2009. Piglets experienced diarrhea and weight loss while the sows experienced reduction in reproduction rate [210]. Two more A(H1N1)pdm09 virus isolates were reported in female swine in Sicily in December 2009 [211] while five isolates of A(H1N1)pdm09 virus were identified in swine at three different locations during 2011–2012 [212].

A novel strain of reassorted H1N2 virus having 99–100% identity through six gene segments with A(H1N1)pdm09 virus along with HA and NA genes similar to H1N2 virus was reported in swine in Mantua Province [213]. Reassorted H1N2 viruses were again detected in 34 piglets during 2013–2014 [214]. 

Seroprevalence of Italian wild boars with one H1N1, ten H1N2, and one H3N2 viruses at two different locations was reported during 2012. On the contrary, active infection was found only in three wild boars whose nasal swabs were positive for the IAV [215]. One more investigation reported active infection of IAV in 12 wild boars while 78 wild boars had IAV antibodies during July–December 2012 [216]. Further molecular and serological investigations detected avian-like H1N1 viruses in Italian wild boars [216]. 

The first complete genome sequence of IDV in Italian swine was retrieved from a symptomatic sow in 2015 which was identified to be closely related to the IDV sequence reported in Oklahoma swine in 2011 [217]. Further IDV prevalence in Italian swine was reported when 14, three and four swine were found positive for IDV antibodies in Veneto, Emilia Romagna and Lombardia regions, respectively during June 2015–May 2016. As a result, swine clinical samples collected during 2013–2014 were investigated retrospectively for IDV prevalence but were reported negative. An extended serological investigation detected IDV antibodies in 364 swine sera samples collected during 2015. These findings suggested that IDV was in circulation in Italian swine population only after 2014 [45].

#### 3.4.9. Spain

Isolation and characterization of 12 H3N2, nine H1N1 and one H1N2 viruses reported the prevalence of influenza viruses for the first time in Spanish swine herds experiencing the respiratory illness and pneumonia during November 2001–April 2004 [218]. More strains of H1N1, H1N2 and H3N2 viruses were isolated, sequenced and characterized in Spanish swine during 2006–2011 [219,220,221]. Interestingly, five H1N1, three H1N2, and four H3N2 virus isolates retrieved between January 2010 and August 2011 had significant similarities with other European isolates which was an evidence of continent-wide transmission of these swine influenza viruses [220]. 

#### 3.4.10. Luxembourg

A molecular investigation reported a negative prevalence of IDV in swine in Luxembourg during 2009 but later successfully detected three IDV positive swine during 2014–2015. Further, the serological investigation confirmed that swine in Luxembourg were free from IDV during 2012 but interestingly, IDV antibodies were detected in 17 swine samples collected during 2014–2015. These observations suggested that IDV was not in circulation in swine in Luxembourg during 2009–2012 but became prevalent at a low frequency later during 2014–2015 [46] which was almost the same time IDV was reported in Italian swine populations [45]. 

#### 3.4.11. The Netherlands

A serological investigation of swine in the Netherlands identified 601 H1N1, 584 H1N2, and 229 H3N2 virus antibodies in 29 swine herds during January–May 1999 [222] with no further evidence of IAV in swine in the country after that. 

#### 3.4.12. Norway

After the 18 swine which were experiencing influenza-like illness were found infected with A(H1N1)pdm09 virus on a Norwegian swine farm in October 2009 the surveillance was expanded to the 39 nearby swine farms which determined that 23 of these farms were positive for the A(H1N1)pdm09 virus. Intriguingly, one human subject at the index farm who had influenza-like symptoms was also found positive for A(H1N1)pdm09 virus. This study suggested that the symptoms first appeared in the human subject at the index farm and later the disease got transmitted to the swine. Hence the findings of this study suggested the reverse zoonosis of the influenza virus from human to pig [223]. 

Further molecular and serological investigations identified 48 more swine herds that were positive for IAV during September 2009–October 2010 [224]. A more comprehensive nation-wide surveillance in Norwegian swine identified 16 A(H1N1)pdm09 virus positive swine herds during 2009 which later increased to 190 swine herds in 2010 [225]. Later 194 more swine were found infected with A(H1N1)pdm09 virus in Norway between April and July 2011 and reported that the IAV infected swine took longer to weigh 100 kg body mass [226]. 

#### 3.4.13. Poland

The first active IAV infection in swine in Poland was reported in 2010 when 21 oral fluid samples collected from three swine farms detected IAV [227]. Soon after, five avian-like H1N1 viruses were reported from the swine lung tissues during 2011–2013 [228]. Later a serological surveillance identified 1212 H1N1, 851 H1N2, 1012 H3N2, and 572 A(H1N1)pdm09 virus antibodies in swine during March 2011–February 2015 [229]. Surprisingly, 34 of these swine had antibodies against all four IAV subtypes i.e., H1N1, H1N2, H3N2, and A(H1N1)pdm09 viruses [229] suggesting the past co-infections. 

#### 3.4.14. Czechoslovakia

The human-like H3N2 virus was isolated from a swine in Czechoslovakia during 1969–1972 [230]; however, no other reports ever appeared from the country in later years. 

#### 3.4.15. Hungary

Complete genome of an H1N1 virus was reported from a Hungarian swine having fever and conjunctivitis in May 2011 [231]. This was the only report of H1N1 virus in the swine in Hungary. 

#### 3.4.16. Multi-National Surveillances in European Countries 

A large-scale investigation across seven European countries reported a high seroprevalence (˃62%) of IAV antibodies in swine populations of Belgium, Germany, Spain, Italy while a relatively lower (˂21.25%) seroprevalence was observed in swine populations of Czech Republic, Poland and Ireland during 2002–2003. Antibodies against H1N1, H1N2, and H3N2 viruses were reported in swine from the European countries under surveillance except Poland where swine had antibodies against only H1N1 virus [232]. 

A virological surveillance across five European countries including Belgium, United Kingdom, Italy, France and Spain reported 169 IAV positive swine during 2006–2008. The H1N1, H1N2, and H3N2 viruses were detected in swine from Belgium, Italy, and Spain while the samples from United Kingdom and France were found infected with H1N1 and H1N2 viruses [233]. 

Briefly, the virological and/or serological prevalence of influenza viruses in European countries (Figure 4D) identified the strains of H1N1, H1N2, H3N2, and A(H1N1)pdm09 viruses in swine populations of the United Kingdom, Ireland, Italy, Germany, France, Norway, Finland, Denmark, Belgium, Spain, Poland, Greece, Hungary, Netherlands, Czech Republic, and Czechoslovakia while the swine in Luxembourg and Italy were found infected with IDV. 

### 3.5. Influenza Viruses in Swine in North America 

#### 3.5.1. Canada

Shortly after a respiratory disease outbreak in swine in Manitoba, an autopsy was done on a dead swine on March 1, 1967. The histopathology confirmed the bronchitis in the deceased swine and a strain of IAV designated as “*S/Manitoba/647/67*” was characterized using IAV antisera [234]. The first report of H1N1 virus in Canadian swine appeared in Quebec during 1980s–1990s when five genotypes of H1N1 virus were identified [235]. Since then several studies have reported H1N1, H1N2 and H3N2 viruses in Canadian swine [236,237,238,239,240,241,242,243]. 

Another study reported nine isolates of swine influenza viruses with an antigenic variant from the sick swine having proliferative pneumonia in Quebec, Canada during 1990–1991 [244]. In a retrospective diagnosis, only one formalin-fixed paraffin embedded swine lung tissue collected during 1991 was found IAV positive with immunohistochemistry. This investigation suggested that immunohistochemistry can be useful in retrospective diagnosis of the swine influenza virus [245]. 

The broncho-intestinal pneumonia in lung tissues of dead swine was reported on a swine farm which exhibited disease symptoms including coughing, weight loss, and labored breathing. Interestingly, before the onset of the disease symptoms, this farm conducted a routine serological surveillance of influenza virus which identified H1N1 virus in only one of the twelve swine samples [246]. 

Following this surveillance, a three-month old swine from the same farm was found positive for avian influenza virus H4N6. The complete genome of this H4N6 virus was reported in 1999. This was the first ever report of an avian-origin H4N6 virus in swine. The proximity of the swine farm to a natural lake where several wild bird species including waterfowls which were reported to visit frequently might be the reason behind the introduction of this avian influenza virus strain to the swine [246]. Later three avian-origin H3N3 influenza virus isolates were recovered from swine in eastern Ontario exhibiting weight loss and coughing during October 2001. On a nearby farm located approximately 30 kms away, another H3N3 virus isolate was recovered from the swine. There was no recorded movement of the swine between these two farms. Since these were avian-origin H3N3 viruses hence the role of birds in transmission cannot be ruled out. Later, on a third farm, where an influenza like disease had been affecting mainly the nursery piglets, an H1N1 virus was recovered in May 2002 [247]. 

Reassortant H1N1 and H1N2 viruses were detected in swine nasal swab or lung tissue samples obtained from three-week old piglets and sows exhibiting typical influenza-like symptoms in Ontario during 2003–2004 [248]. First triple-reassortant (avian/classical swine/human triple-reassortant) H3N2 viruses from four swine and one human nasal samples were identified in Ontario during 2005. The phylogenetic analysis determined that all the virus sequences were 100% identical to each other which apparently emerged from triple-reassortant H3N2 viruses reported in US based swine in 1988 [249]. One more report of triple-reassortant H3N2 (trH3N2) viruses appeared on the swine farms located in Saint-Hyacinthe, Assomption and Saint-Foy during early 2009. The trH3N2 viruses identified in this study were determined to be closely related to North American/Canadian trH3N2 viruses reported earlier [250]. Later A(H1N1)pdm09 and H1N1 viruses having internal genes of triple reassortant H3N2 virus were reported in swine in four provinces including Manitoba, Alberta, Saskatchewan and Quebec during 2009 [251].

The first evidence of A(H1N1)pdm09 virus in Canadian swine appeared in 2009 after the human workers at a swine farm developed influenza-like illness. The investigation identified that two farm workers along with 56 swine were positive for the A(H1N1)pdm09 virus. Transmission of A(H1N1)pdm09 virus from human to swine was suggested [252]. The same year 17 more swine were detected with A(H1N1)pdm09 virus after a respiratory disease outbreak hit the Alberta swine farms [253]. 

A reverse zoonotic transmission of A(H1N1)pdm09 virus to swine from a human subject who visited Mexico and returned to the swine farm was reported in April 2009. As a result, ten swine having severe disease were sacrificed for necropsy which identified lesions in the bronchioles corresponding to the influenza virus disease. Virus isolation and sequencing identified the A(H1N1)pdm09 virus. Additionally, A(H1N1)pdm09 virus was identified in two more human subjects who were exposed to the swine hence indicated the occurrence of zoonoses on the swine farm [254]. 

Later during summer 2009, ten more A(H1N1)pdm09 viruses from five swine herds in Manitoba were reported. Virus shedding was observed up to 20 days post-infection after the appearance of the clinical symptoms in swine [255]. This observation was in agreement of a previous report which documented the experimental infection of swine in the laboratory and determined that virus shedding occurs until 11th day after appearance of the clinical symptoms [256]. Another investigation reported nine A(H1N1)pdm09 and four H3N2 viruses after an influenza-like disease outbreak on a Quebec based swine farm in December 2010 [257]. 

The effect of microclimatic conditions on the transmission dynamics of swine IAV in the barns was studied which observed that high relative humidity in the environment during summer months suppresses the aerosol transmission of the droplets which in turn decreases the transmission of IAV [240]. The high relative humidity in the environment would facilitate the generation of larger droplets which do not tend to shrink easily and hence are less likely to be aerosol transmitted to a longer distance as they fall on the ground quickly after their formation [240,258]. As a result, a lower transmission of IAV is observed usually during the summer months. On the contrary, the IAV transmission increases during winter months when relative humidity is relatively lower [258]. 

#### 3.5.2. United States

The IAV was first isolated from the nasal discharge of a swine in the United States in 1931 [34] and from the human in 1933 [35]. The first report of human-origin IAV in swine appeared in the United States on 24 May 1937 after an unexpected result was observed when the serum sample of a sick swine obtained from a State Prison Farm located in New Jersey neutralized the antibodies of human influenza virus. A series of investigations made a strikingly new observation that swine had suffered from a human strain of influenza virus [259]. 

Serological investigations conducted during 1950s suggested that the weight loss and mortalities among swine were due to swine influenza viruses [260,261]. Swine influenza viruses were isolated from febrile swine at nine occasions during 1965–1968 in Wisconsin and Nebraska [262]. Additionally, swine influenza antibodies were also detected in swine sera samples collected from six farms [262]. A virological surveillance conducted in Memphis, Tennessee and Madison, Wisconsin during May 1976 to June 1977 successfully isolated 478 influenza viruses from swine nasal swabs collected at abattoirs; approximately 300 of which were characterized to be swine H1N1 viruses. Additionally, the serological surveillance identified that 21% of the 9400 swine sera samples had swine H1N1 virus antibodies [263]. A small percentage (1.4%) of swine sera samples were found positive for the swine H3N2 viruses which was further confirmed by virus isolation [263]. Interestingly, this study identified inter-species transmission of swine influenza viruses between human and swine [263].

A novel swine-origin H1N1 virus termed as “*A/New Jersey/76 (Hsw1N1)*” was detected at Fort Dix Army training camp in New Jersey in January 1976. The outbreak was localized and was limited to Fort Dix only. As a result, 230 soldiers were found infected with this novel virus; 13 of which had severe respiratory disease with one death due to viral pneumonia [264,265,266]. Since this novel swine-origin H1N1 virus quickly disappeared from Fort Dix hence the epidemiology and the origin of the disease could not be ascertained [264].

The H1N1 and H3N2 virus antibodies were detected in swine sera collected from an abattoir in North-West United States. Interestingly, a higher IAV seroprevalence was observed during the Fall and early winter months. Virus isolation and sequencing identified that the H1N1 viruses were closely related to the classical H1 swine influenza virus [267]. Classical swine-like H1N1 and triple-reassortant H3N2 viruses were identified in swine samples collected across 23 states in the USA during 1998–1999 [268]. 

The Minnesota Veterinary Diagnostic Laboratory (MVDL) detected large number of H1N1, H1N2 and H3N2 subtypes of IAV in swine samples during 1998–2001 and again during 2007–2009. Interestingly, some of the samples were co-infected with H1N1 and H3N2 viruses [269,270,271]. A second-generation reassortant H1N2 virus having genes from a reassortant H3N2 and classical H1 swine influenza viruses was obtained from the lung tissue samples of a dead sow at an Indiana swine farm in November 1999 [272]. 

A novel subtype of H3N1 virus termed as “*A/Swine/Minnesota/00395/2004 (H3N1)*” was identified during a severe respiratory disease outbreak on a swine farm in Minnesota in October 2004. Sequencing observed that the HA gene of this strain was closely related to swine influenza H3N2 virus while the NA gene was related to classical H1N1 virus which suggested that the novel H3N1 virus emerged due to reassortment between H1N1 and H3N2 viruses in the Midwest United States [273]. Further an H2N3 subtype of IAV which may have emerged as a result of a reassortment between avian and swine influenza viruses was identified on a commercial swine farm in Minnesota in April 2006 and again in September 2006 [274]. 

The first evidence of A(H1N1)pdm09 virus infection in US swine appeared when four A(H1N1)pdm09 and one triple-reassortant H1N2 viruses were identified and characterized in the exhibition swine in the states of Minnesota and South Dakota in 2009 [275]. During last ten years, a large number of H1N1, H1N2, H3N2, A(H1N1)pdm09 along with reassortant IAV subtypes have been reported in the US swine populations [243,276,277,278,279,280,281,282,283,284,285,286,287,288,289]. 

The United States has a large feral swine population which is considered a reservoir of H1N1 and H3N2 viruses [290]. The swine-like H1N1, avian-like H1N1, swine-like H1N2, swine-like H3N2, human-like H3N2, A(H1N1)pdm09 along with avian-like H6N2 and H7N2 viruses were identified in feral swine samples collected across 35 states in the USA between October 2009–September 2013 [291]. 

Histological examination of the lung tissues obtained from two backyard piglets suffering from pneumonia and weight loss in Colorado in November 2010 suggested that the piglets were infected with swine influenza virus which were later confirmed to be infected with IAV subtype A(H1N1)pdm09 virus. Since the piglets were raised at the house of a pharmacist hence a possible human to swine transmission was speculated given the possibility of an occupational exposure of the pharmacist to the A(H1N1)pdm09 virus at the pharmacy [292]. 

The first report of IBV infection in swine appeared when swine in the Midwest United States were found infected with IBV lineages of Yagamata/B and Victoria/B [37]. This was a new finding because initially IBV was thought to have a host range limited to human, pheasants, horses and seal [1,2,3,4]. 

A novel strain of swine influenza virus was detected in Oklahoma swine exhibiting influenza-like symptoms in April 2011. The nasal swab samples taken from the swine were negative for the IAV infection. Hence the virus isolation was attempted in swine testicle cells; the cells in culture showed influenza-like cytopathic effects by third day. Electron microscopic observations revealed particles typical of a virus of Orthomyxoviridae family, but the RT-PCR was negative for the IBV and ICV. After ultracentrifugation was used for virus isolation, the genome of the virus was sequenced using Ion Torrent sequencing. The genome sequence analysis along with genetic and biochemical investigations revealed that the isolated virus was a novel Orthomyxovirus having 50% overall identity at amino acid level with human influenza C virus [43]. Since this novel virus was genetically and antigenically distinct from ICV therefore, later was proposed to be categorized as a new genus of Orthomyxoviridae family which was later accepted as influenza D virus (IDV) [5]. 

Later, two feral swine which were shot dead in a cotton field in Texas in June 2011 were found infected with A(H1N1)pdm09 virus. The significant identity of A(H1N1)pdm09 virus isolated from these two feral swine with human A(H1N1)pdm09 virus suggested a possible transmission between human and the feral swine [290]. Another study reported seroprevalence of H3N2 virus in one feral swine from Mississippi and in five feral swine from the state of California in 2005 but a negative seroprevalence was reported in the feral swine samples obtained from the states of Florida, Oklahoma and Missouri. Additionally, the seroprevalence of IAV was reported in feral swine from Texas where a total of 68 out of 472 feral swine sera were found positive for H3N2 and H1N1 viruses [293]. 

Another investigation detected H3N2 virus RNA in only one feral swine from a pool of samples collected across 31 states in the USA during 2011–2012 which indicated a negligible active influenza infection in US feral swine population. On the contrary, ELISA identified IAV antibodies in 182 feral swine samples while the serological subtyping identified H3N2 virus antibodies in 76 feral swine samples collected from 19 states which indicated a significant past exposure of US feral swine to the H3N2 virus [294]. Further, seroprevalence of IDV was reported in 49 feral swine samples collected from Oklahoma, Texas, Hawaii and North Carolina during October 2012–September 2013 which provided the first evidence of past IDV infections in US feral swine [295]. 

A study investigating virus shedding in nursery piglets found that all 81 piglets under investigation were shedding H3N2 virus starting seventh day of arrival into the barns until 29th day. Shedding was still observed in some piglets until 39th day [296]. Interestingly, 48 of these nursery piglets were also identified shedding H1N1 virus starting at the third day of arrival into the barns until 41st day over a 53-day observation period [296]. This was the new information which identified that young nursery piglets could get infected with IAV. 

The oral fluid samples collected from 25 neonatal piglets at four Oklahoma based swine farms during May–August 2014 [297] were found infected with different IAV subtypes including H1, N1, H3, and N2. This study supported the use of swine oral fluid samples in IAV diagnostics [285]. The swine oral fluid samples were also collected in North and South Carolina during June to August 2014 using the cotton rope hanging method [298]. In this method of sampling, swine are encouraged to chew the rope, as a result, saliva accumulates on the rope which is later squeezed to collect the sample aseptically. One of the benefits of this method of sampling is that each sample does not represent an individual swine but rather represents multiple swine that chewed the rope while hanging inside the pen [298]. Another benefit of this sampling method is that swine oral samples may contain contaminants like feed and feces but this method minimizes the chances of such contaminations in the sample [299]. 

Another investigation carried out metagenomic sequencing of swine nasal and rectal swabs obtained from apparently healthy swine which identified 11 IAV positive swine at three abattoirs and a buying station in USA in August 2015 [300]. 

In a striking observation, an avian-lineage H4N6 virus was isolated and sequenced from 7–8-month-old gilts on a Missouri based swine farm in December 2015 [301]. The investigators collected more samples at different time points for next few months at the same farm to assess the transmission of H4N6 virus among swine. No other samples were found positive for the H4N6 virus which suggested that the H4N6 virus did not transmit from swine-to-swine and therefore disappeared from the index farm. Interestingly, this extended study identified three H1N1 viruses infecting swine [301]. 

One large-scale study identified that 23 percent (2 947/12,814) of the swine samples were positive for the IAV in Mid-West United States between July 2011–March 2017, however, sequencing could identify only 173 H1 and H3 subtypes among positive samples [302]. A human to swine transmission of IAV was suggested when two human-like H3N2 virus isolates were identified from an Oklahoma based swine farm in 2017 which had high similarity with the human-like H3N2 viruses reported earlier from Baltimore [303]. 

#### 3.5.3. Mexico

Maya people represent ethnolinguistic groups in South and Central America. The practice of household swine keeping put the Maya people at high risk of contracting the swine influenza viruses. Thirty-one sera samples collected from the Maya people in Mexico were identified having antibodies against H1N1 and H3N2 viruses while 93 other sera had antibodies against the H3 subtype of IAV, representing a past exposure to these viruses [304]. However, this study did not include swine samples for investigation but since swine were household animals in their backyard hence the IAV seroprevalence of the Maya people could be because of a past transmission of these viruses from the backyard swine [304]. 

A retrospective study identified antibodies against swine-like H1N1, A(H1N1)pdm09, H3N2, and human-like H1N1 viruses in backyard swine in Mexico between 2000 to 2009. This investigation retrospectively determined that the classical-swine H1N1 virus was most widely present in Mexican swine before the 2009 influenza pandemic [305]. Further, a significant number of swine experiencing respiratory illness had H1N1 or H3N2 virus antibodies in commercial piggeries in Sonora Province of Mexico during October 2008–March 2009. The molecular diagnostics and subtyping determined four H1 and two H3 viruses while 19 other IAV positive samples could not be subtyped given the low viral load [306]. 

During the influenza virus pandemic in Mexico in 2009, A(H1N1)pdm09 virus was first identified in a single swine nasal swab. Additionally, H3N2, A(H1N1)pdm09 and IBV viruses were detected in four symptomatic humans [307]. The A(H1N1)pdm09 virus isolate retrieved from the swine was believed to be the first from the sister lineage of the pandemic influenza virus isolates reported in Mexico [307]. 

Further 59 IAV isolates were retrieved from Mexican swine having respiratory illness during 2010–2014. Intriguingly, this study identified 13 reassorted genotypes of IAV in Mexican swine [308]. This investigation also reported that IAV introduction into Mexican swine may have occurred through three different routes; human to swine transmission; reassortment between human-like H3N2 and A(H1N1)pdm09 virus; and through the long-distance movement of the swine from USA and Europe. A periodic introduction of IAV in Mexican swine occurred with the import of American and European swine to Mexico over two decades in 1980s and 1990s before the 2009 influenza pandemic [33]. 

Fifty-eight IAV whole genome sequences were retrieved from Mexican swine during 2010–2014. Genome sequence analysis identified classical H1N1, H3N2, and A(H1N1)pdm09 viruses. Interestingly, the data obtained in this study suggested independent evolution of IAV in the Mexican swine population in different regions of the country. Phylogeny determined that Mexico City was the source of the 2009 influenza pandemic which erupted during March–May 2009 [33]. Later a reassortant H1N2 virus which had the genes from human and swine influenza viruses was isolated and sequenced from a swine in November 2014 [309]. 

#### 3.5.4. Guatemala

The molecular diagnostics identified a total of 104 IAV positive commercial and backyard swine in Guatemala during 2010–2011 which resulted into three A(H1N1)pdm09 and one H3N2 virus isolates [310]. 

#### 3.5.5. Cuba

The first report of A(H1N1)pdm09 virus in commercial piggeries in Cuba appeared in November 2010 when 24 swine were found positive for A(H1N1)pdm09 virus across five swine farms [311]. Further, five more IAV positive swine were detected in Pinar del Rio province of Western Cuba having respiratory illness and interstitial pneumonia. However only one IAV positive sample could be successfully subtyped as A(H1N1)pdm09 virus having reassorted internal genes, all except the NA gene [312].

#### 3.5.6. Trinidad and Tobago

In a more recent investigation, a high seroprevalence of IAV (114/309) was detected in swine in Trinidad and Tobago which later identified H3N2 and A(H1N1)pdm09 viruses in swine [313]. 

In summary, the H1N1, H1N2, H3N2, and A(H1N1)pdm09 viruses were reported in North American swine population. Interestingly, the avian influenza virus strain H4N6 was detected in US based swine while H3N3 and H4N6 were identified in the Canadian swine and H5N2 was reported in the Mexican swine in 2018 (Figure 4E). Mexico City was identified to be the origin of 2009 influenza pandemic. It was also ascertained that A(H1N1)pdm09 virus was present in Mexican swine well before 2009 pandemic erupted. 

### 3.6. South America

#### 3.6.1. Argentina

After influenza virus outbreak hit a swine farm in Buenos Aires in November 2008, one of the five dead swine were diagnosed with viral pneumonia through immunohistochemistry. A full genome of H3N2 virus sharing 96–98% nucleotide sequence identity with H3N2 viruses reported in North America during 2000–2003 was recovered from the swine [314]. 

An H1N1 virus was reported from a swine after a swine farm manager along with his spouse experienced influenza-like symptoms few days before the outbreak erupted in the swine at a Buenos Aires based farm in June 2009. The influenza disease symptoms lasted for about a week in nursery piglets. Immunohistochemistry identified necrotizing bronchiolitis in four of the swine post-mortem samples while one sample had severe inflammation in the bronchiolar epithelia. The serological investigation detected IAV antibodies in most of the sera samples collected after 15 days of onset of clinical symptoms however the active infection was reduced to only six swine [315]. 

The third investigation carried out histopathology which identified lung lesions compatible to the influenza virus infection in nine swine necropsy samples at a Buenos Aires based swine farm in October 2009 and later in eight swine necropsy samples originated from a Santa Fe based farm in May 2010. The swine at Buenos Aires farm were found infected with H1N1 virus while the swine at the Santa Fe farm retrieved one H1N2 and three human-like reassortant A(H1N1)pdm09 virus isolates which had triple reassortant internal genes. This was the first report of human-like reassortant A(H1N1)pdm09 virus in swine in Argentina [316]. Later two more investigations using histopathology, immunohistochemistry, serology, and molecular analyses reported H1N2, H3N2, and reassortant H3N2 viruses with A(H1N1)pdm09 internal genes in swine in Argentina during 2011–2012 [317,318]. 

#### 3.6.2. Brazil

Several H1N1, H1N2, H3N2, human-like H1N1, and A(H1N1)pdm09 viruses have been identified in Brazilian swine from the Minas Gerais, Parana, Rio Grande do Sul and Sao Paulo provinces in Brazil during and after 2009 [319,320,321,322,323,324,325]. A technician who visited a Minas Gerais swine farm experiencing influenza outbreak developed similar respiratory disease symptoms. The nasal swab sample was obtained from the technician, as a result, one A(H1N1)pdm09 virus was isolated which was closely related to the A(H1N1)pdm09 viruses reported from the swine herd in the Minas Gerais which was recently visited by the technician. Hence it was concluded that a zoonotic transmission from swine to the technician occurred at the Minas Gerais swine farm [326]. 

An immunohistochemical investigation demonstrated microscopic lesions suggesting broncho-interstitial pneumonia in the lung tissues of four severely sick piglets at a swine farm located in Parana province in February 2011. The A(H1N1)pdm09 viruses were isolated from two piglets. Additionally, a novel reassortant H1N2 virus was also recovered [327]. One more investigation identified that A(H1N1)pdm09 virus was the most prevalent IAV subtype in sows. The co-infections of sows with A(H1N1)pdm09, H1N2, or H3N2 subtypes were also documented in Rio Grande do Sul province. These findings were noteworthy because the coinfections may trigger reassortments and thus may facilitate emergence of novel strains of IAV [328]. Later two more H1N2 viruses were isolated and characterized from swine in Rio Grande do Sul province during 2013. The sequences of both the isolates had high nucleotide similarity to each other in different genome segments in the range of 98.9% to 100% which suggested a common source of origin of both isolates [329]. 

#### 3.6.3. Colombia

The A(H1N1)pdm09 virus was identified in seven swine farms in Colombia during 2008–2009 [330]. 

#### 3.6.4. Peru

The A(H1N1)pdm09 virus antibodies were detected in 110 backyard swine in Peru during March 2009–October 2011. Total four A(H1N1)pdm09 virus isolates were retrieved and sequenced which determined that there were at least two separate events of A(H1N1)pdm09 virus transmission from human to backyard swine in Peru [331]. 

#### 3.6.5. Chile

The backyard productive systems (BPS) for raising swine, cattle, and poultry are popular in Chile. A molecular investigation reported a negative active IAV infection across 113 BPS units within ten counties in Chile during 2012–2014 but the serological investigation detected IAV antibodies in swine at two BPS units which suggested a past exposure of swine to the IAV [332]. Interestingly, the HA gene sequence of an H12 virus was obtained from a domestic Muscovy duck at one of the BPS which appeared to have originated from a wild bird. This suggested a spillover of the IAV from wild reservoir to the domestic poultry [332]. 

Another study reported the prevalence of H1N2 virus in swine reared at 40 different BPS having poultry and swine in El Yali wetland during 2013–2014 [333]. One more study identified four swine sera samples (4/64; 6.3%) that were found positive for IAV antibodies collected from different BPS in Central Chile. One pool of swine nasal swab samples (1/39; 2.6%) was also detected IAV positive with real-time RT-PCR. Interestingly, 7.9% chicken, 4.3% ducks and 11.1% geese samples collected from 329 BPS in Central Chile also had active IAV infections. The breeding practice of poultry and swine in the BPS was determined to be a major risk factor for IAV transmission [334]. 

Briefly, the IAV strains of H1N1, H1N2, H3N2, and A(H1N1)pdm09 viruses have been reported from the swine in Argentina and Brazil while A(H1N1)pdm09 virus was reported in swine in Colombia and Peru. Swine in Chile were found infected with H1N2 virus (Figure 4F).

In summary, total 281 research articles were identified which reported several influenza viruses in swine populations globally. The highest number of studies were reported from Asia (*n* = 107), followed by North America (*n* = 76), Europe (*n* = 55), South America (*n* = 21), Africa (*n* = 18) and Australia (*n* = 4). The highest number of reports per country were documented in United States (n = 40) followed by China (*n* = 39) and Canada (*n* = 24). Until February 2020, influenza viruses have been reported from 53 countries worldwide. Four subtypes of IAV including H1N1, H1N2, H3N2, and A(H1N1)pdm09 viruses were most frequently detected in swine populations (Table 1). 

Most of the large-scale studies used serological investigations including ELISA, hemagglutinin inhibition (HI), neuraminidase inhibition (NI), virus neutralization (VN), or microneutralization (MN) assays for the determination of the seroprevalence and subtyping of the influenza viruses in swine. Several investigations used virus isolation for the confirmation and subtyping of IAV. Most of the virological investigations used one-step real-time RT-PCR and/or reverse-transcription PCR for influenza virus detection and subtyping. Sanger sequencing or next-generation sequencing using MiSeq or Ion Torrent sequencing successfully generated the influenza virus sequences from the swine samples for epidemiological interpretations. Histological examinations including immunohistochemistry or immunofluorescence were used to examine the swine lung or other internal organ tissue samples for the influenza virus diagnostics (Table 2). 

## 4. Discussion 

As of February 2020, influenza viruses have been identified and reported in swine from 53 countries worldwide (Table 1; Figure 3). The influenza viruses have been detected in different sample types including swine sera, nasal, tracheal, oropharyngeal, nasopharyngeal swabs as well as oral fluids collected from the live swine. Nasal and snout wipes, lung homogenates and fecal slurry samples were also used. Additionally, the lung as well as other internal organ tissues (Table 2) obtained from either dead or sacrificed swine have also been used for the detection of IAV symptoms i.e., lesions in lungs, pneumonia, bronchitis, bronchiolitis etc.

Various methods have been used for the detection of influenza viruses in swine samples depending on the sample type, sample numbers and objective of the study. Virus isolation methods, using either MDCK, Caco-2, HRT18, or swine testicle cells or the pathogen free embryonated chicken eggs, although considered the gold standard [335,336,337] have largely been taken over lately by the sequencing approaches which tend to provide a considerably faster identification of the IAV subtypes. The additional benefit of sequencing over virus isolation is that the sequences would be useful for analyzing the influenza virus outbreak clusters [338], virus evolution or reassortment [339] using phylogenetic analyses in different gene segments. A recent study reported that next-generation sequencing can be useful in the influenza virus diagnosis and for the identification of the novel virulence markers and drug resistance [340]. 

Most of the studies have used real-time RT-PCR with matrix-gene specific oligonucleotide primers and TaqMan probe for IAV detection [308,341,342]. The conserved sequences of the matrix-gene specific primers can detect any IAV subtype in the swine samples [343]. Most of the studies used subtype-specific real-time RT-PCR for the IAV subtyping, however, few studies opted for the conventional approach of reverse-transcription PCR followed by Sanger sequencing for amplification of the HA and/or NA genes for retrieving the sequences for phylogenetic analyses to identify the subtypes. Although the real-time RT-PCR is a powerful and rapid tool for the subtyping of IAV strains, it is more expensive than reverse-transcription PCR. A few studies have reported reverse-transcription PCR based amplification of all the eight gene segments of IAV to generate the whole genome sequences [33,91,128,231] but in most cases, the whole genome sequences were generated using MiSeq next-generation sequencing approach [150,214,325,329]. A great advantage of this sequencing approach is that it can identify novel influenza viruses in the swine samples [80,90,301]. 

Most of the serological investigations used one or more methods for influenza virus detection and subtyping in the swine samples e.g., ELISA, HI, NI, MN, or VN assays. The serological methods are useful in large-scale surveillances for screening large number of samples in a limited time. However, the molecular detection assays are more reliable than the serological methods given the higher sensitivity, but the serological assays are rapid and affordable hence are preferred for large-scale surveillance studies. The molecular and serological investigations report either active infections (viral RNA) or past exposures (antibodies) in swine samples, respectively. The molecular detection approaches followed by sequencing are largely used for the research focusing on the influenza virus epidemiology [73,78,82,87,94,128,146,150]. 

Several studies used histological examinations e.g., immunohistochemistry or immunofluorescence to identify the IAV symptoms in lung or other internal organs of either dead or severely sick swine sacrificed for the investigations [130,189,315,318]. Immunohistochemistry provides a rapid and affordable diagnosis of the influenza virus disease using swine tissue samples [344]. One major benefit of immunohistochemistry is that it can be used in the retrospective analysis of the archived tissue samples [245]. 

**Figure 5:**
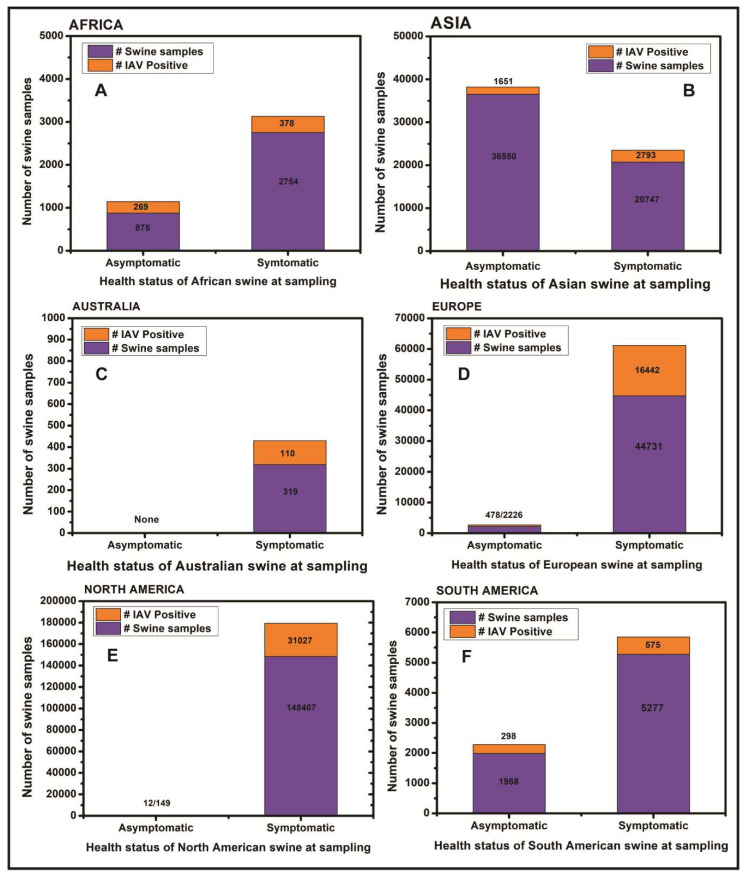
Health status of the swine at the time of sampling in (**A**) Africa, (**B**) Asia, (**C**) Australia, (**D**) Europe, (**E**) North America, and (**F**) South America. The asymptomatic swine were apparently healthy with no clinical symptoms while the symptomatic swine had mild to severe influenza-like disease symptoms. Most of the swine in North America, Australia, Europe, South America, and Africa represented influenza-like illness at sampling while most of the Asian swine were apparently healthy at the time of sampling. It should be noted that asymptomatic cases may not be detected if that was not an aim of the surveillance study. These graphs do not represent the severity of disease as the swine populations and the objectives of surveillances may vary among countries.

A large number of investigations have reported sub-clinical influenza virus infections in asymptomatic (apparently healthy) swine [78,103,150,161,171,278,300], indicating that influenza infections can go undetected while the swine may be shedding virus and hence may infect other swine and farm workers in contact [183]. Intriguingly, most of the swine samples processed in Australia, Europe, and North America were obtained from the symptomatic swine while most of the Chinese swine samples were collected from asymptomatic swine (Figure 5).

Symptomatic swine may exhibit mild or severe influenza like symptoms [59,202,252], including fever, coughing, sneezing, pneumonia, bronchitis, reduced appetite, diarrhea, nasal and/or ocular discharge, conjunctivitis, weakness, anorexia, prostration, weight loss, abortion in sows, and mortality in some cases [89,224,231,239,248,282,318]. Most studies where the swine were severely infected reported reduced appetite and weight loss [129,246,247,292]. Due to IAV infection, the swine takes longer to weigh 100 kg body mass [226], hence the IAV disease burden affects the swine farmers economically. 

Varying rates of mortality of swine due to IAV infections were reported from around the world ranging from 0.5% to 30% [138,206,213,218,221,273,296,327]. This wide difference in mortality rate could be due to novel virus strains emerged through reassortments within the swine [273,315,327] or inter-species transmission, e.g., avian to swine transmission, resulting into severe disease outbreaks [27,80,119,133]. One example of the emergence of a novel influenza virus strain is the emergence of A(H1N1)pdm09 strain due to reassortment between avian and swine IAV strains in swine which resulted into 2009 influenza pandemic [33]. Additionally, the emergence of IAV subtype H1N2 is another classic example of influenza virus reassortment which resulted into severe disease outbreaks in Japanese and Korean swine populations during 1990s and 2000s [27,121,140]. 

Strains of IAV can infect the swine of any age group; piglets as young as one week may become infected with IAV naturally. Interestingly, a study in Denmark observed a piglet as young as just three days was infected with IAV despite having maternally derived IAV antibodies [183], suggesting that the infection might have occurred from the infected sow which was shedding the virus [183]. However, the symptoms of the influenza-like illness in swine may last only for one week but the virus shedding may still persist until 41 days after appearance of the influenza-like symptoms [183,296]. This phenomenon may have serious implications in influenza virus spill over to the non-infected swine as well as to the exposed farm workers due to prolonged virus shedding. Three other studies observed virus shedding in swine and reported that the virus shedding may persist until the 11 day [256], 20 day [255], or 29 day [296] after onset of the clinical symptoms in swine. This variation in the duration of the virus shedding might be strain dependent, which needs to be further investigated. 

A higher rate of virus shedding and IAV prevalence was reported during the fall and early winter months than summer season because the high relative humidity present in the environment during summer decreases the transmission of influenza virus [267]. The high relative humidity in summer facilitates the generation of larger droplets which are less likely to be aerosol transmitted to a longer distance as they tend to fall on the ground quickly after their formation [240,258]. 

**Figure 6:**
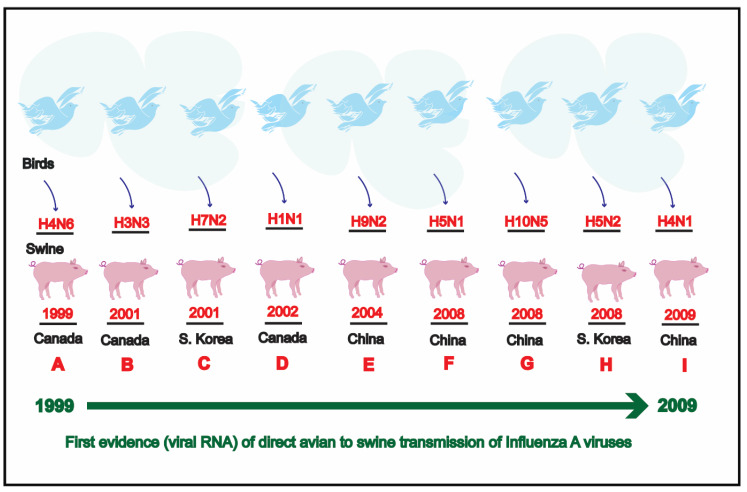
The first evidence of direct avian to swine transmission of avian-origin IAV strains. Viral RNA was detected through real-time RT-PCR and the partial or whole genomes were sequenced for the characterization of avian-origin IAV strains retrieved from the swine for the first time in Canada, South Korea and China.

Several cases of inter-species transmission were identified which documented transmission of IAV between human and swine or between birds and swine. The occurrence of the avian influenza virus strains in swine in China (H5N1, H9N2, H4N1, H4N6, H5N3, H10N5, H4N8, H6N6, H7N9), United States (H4N6, H6N2, H7N2), Canada (H4N6, H3N3), South Korea (H7N2, H5N2), Nigeria (H5N1), and Egypt (H5N1, H5N2, H9N2 ) serve as the evidence of interspecies transmission of avian influenza viruses to swine. The first evidence of avian influenza virus active infection in swine appeared in 1999 in Canada when H4N6 virus was isolated from a swine. Later several other avian-origin IAV strains were detected and sequenced in swine in China, Canada, and South Korea (Figure 6). 

Various studies have spotted wild birds visiting the swine farms or in the vicinity which suggested that wild birds may have served as the carriers for the introduction of the different avian-origin IAV subtypes to the swine populations [59,80,100,246,332]. The highest number of avian-origin IAV strains were reported in Chinese swine which shows frequent avian-swine interaction in China, a country that has historically been an epicenter for influenza virus disease [69].

Egypt is recognized as a “hot spot” for the influenza virus reassortment due to its geographical location [345]. The role of migratory wild birds in the introduction of avian influenza in Egypt has been already established [346,347], and the highly pathogenic strains of the IAV have previously been detected in migratory birds in Egypt [348]. Since migratory wild birds were reported to harbor in the vicinity of Cairo [60] therefore, the probability of the migratory bird–swine interaction in the regions remain high which very well explains the occurrence of highly and low pathogenic strains of avian-origin IAV in swine in Cairo, Egypt. Given the “mixing vessel” nature of the swine, the occurrence of avian-origin IAV strains in swine is alarming in terms of IAV reassortment and evolution which may trigger the emergence of novel IAV strains of pandemic potential in the future. 

Further, the multiple reports of double or triple reassortant IAV strains in swine are evidence that IAV co-infections may facilitate the antigenic diversity of the influenza viruses; and as a result, new HA and NA subtypes of IAV may be continually added to the existing 18 HA and 11 NA subtypes in the future. Intriguingly, the frequency of the occurrence of double or triple-reassortant IAV strains in swine has dramatically increased in the recent decades [76,81,87,109,161,250,268,278]. One unique example of the reassortment and evolution of the pandemic strain of IAV in swine was the emergence of A(H1N1)pdm09 virus in swine in Mexico which evolved due to the reassortment between avian and swine IAV strains [33]. 

**Figure 7:**
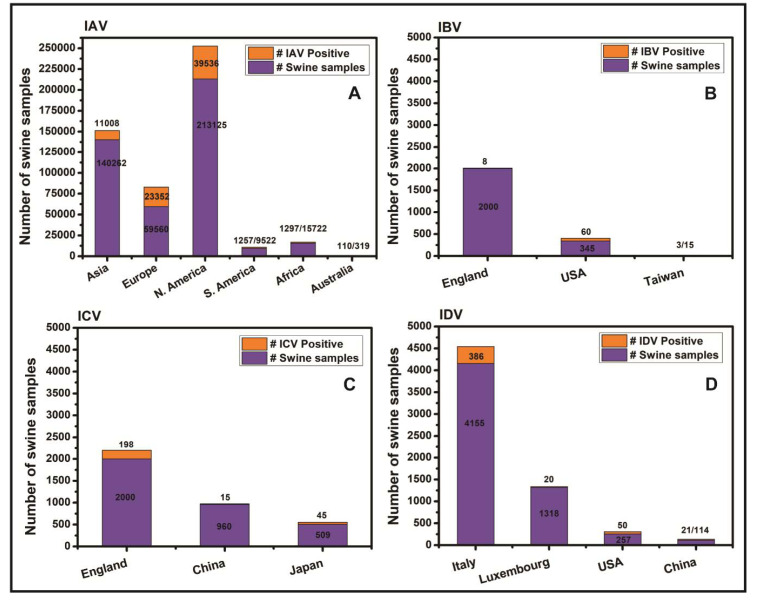
Prevalence of all the four types of influenza viruses; (**A**) IAV, (**B**) IBV, (**C**) ICV and (**D**) IDV in swine populations worldwide. The highest IAV prevalence was reported in North American swine population followed by Europe and Asia. A significantly lower prevalence of IBV, ICV, and IDV was detected in certain Asian, European and North American countries.

While an overwhelming majority of investigations reported IAV in the swine across the world (Figure 7A), there were only a few reports which documented either active infections or past exposures of the swine to the influenza virus types IBV [37,171,186,277] (Figure 7B), ICV [38,117,186] (Figure 7C) or IDV [5,43,44,45,46,217] (Figure 7D). A low prevalence of IBV was observed in swine given that only one study reported the IBV antibodies in swine samples in England during 1991–1992 [186] with no evidence of further spill over to other European countries. The active infection of IBV was later reported in US swine when two IBV isolates were obtained in 2009. A recent study from Taiwan reported three strains of the Victoria/B lineage of IBV in naturally infected swine in 2014 [171], again there was no further report of dissemination to nearby Asian countries. The IBV infected swine were apparently healthy with no signs of influenza disease. 

The first report of ICV appeared in Chinese swine after the virus was isolated from apparently healthy swine in 1981 in a routine diagnostic procedure at an abattoir in Beijing [38]. Later ICV seroprevalence was reported in English and Japanese swine during 1980s–1990s [117,186] with no further evidence of circulation anymore thereafter. 

The IDV was first detected and characterized in 2011 in Oklahoma based swine in the United States which appeared to have made a species jump from cattle to swine [5,43]. Interestingly, a complete IDV genome was retrieved from a symptomatic sow in Italy in 2015 which was found closely related to the IDV genome reported in 2011 from Oklahoma, USA [217]. This might have happened due to the trade of the cattle or swine between Italy and the United States. A recent study from China identified IDV sequences which shared a high similarity (99%–100%) with the IDV sequences reported earlier from the cattle in China [102] which was another evidence of bovine to swine transmission of IDV. The IDV has been in circulation in swine in the current decade with reports emerging from swine in Italy, Luxembourg, China and the United States. 

**Figure 8:**
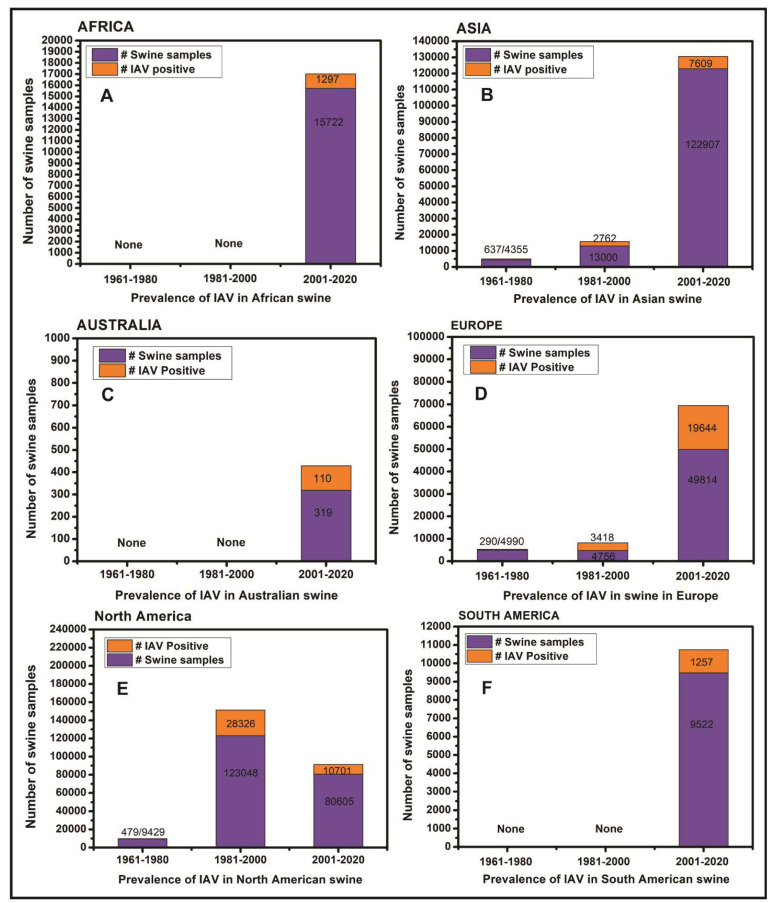
Historical progression (1961–2020) of IAV prevalence in swine populations in (**A**) Africa, (**B**) Asia, (**C**) Australia, (**D**) Europe, (**E**) North America, and (**F**) South America. An increasing IAV prevalence was observed in all the continents except North America where a smaller number of cases were observed during last two decades than 1981–2000.

In summary, IAV was first isolated from a swine in USA in 1930 [34,349] and later antibodies for the human influenza viruses were reported in swine at the State Prison of New Jersey in 1937 [259]. More IAV outbreaks and cases in swine in North America were reported during 1981–2000; the frequency has now dramatically fallen in the last two decades (Figure 8). This might be due to improved swine influenza surveillance and vaccination in North America in recent decades. The H1N1, H1N2, H3N2, and A(H1N1)pdm09 viruses were reported from the commercial, backyard, exhibition, feral swine and wild boars in the United States. 

As of February 2020, the highest number of reports of influenza virus infections in swine in a country were documented in the United States (*n* = 40) followed by China (*n* = 39) and Canada (*n* = 24). The highest number of IAV positive swine samples were reported in the United States (36128/200384) followed by China (5031/90760). One of the factors behind the higher number of IAV cases in swine in the United States compared to China would be related to the disease symptoms. A majority of the North American swine samples that were screened for the IAV infections had either mild or severe symptoms of influenza-like illness which would have made it visually easier to identify IAV infected swine in the United States. On the contrary, a smaller number of Chinese swine exhibited influenza-like disease symptoms while a large proportion of the Chinese swine population appeared to have sub-clinical infections with no symptoms. This would have made it difficult for identifying the influenza virus infected swine during surveillances in China. 

The first report of IDV in 2011 in Oklahoma swine reflected the antigenic diversity and evolution of influenza viruses in the US swine. However, the recent influenza virus disease prevalence in North American swine appeared to have declined, nevertheless, given the large swine population of the continent, the surveillance should continue to track the influenza virus diversity and evolution.

The first serological evidence of IAV in European swine was documented from the Czechoslovakia during 1969–1972, but the first H1N1 virus in European swine was isolated in Belgium in 1979 which was apparently transmitted from wild ducks in Germany to the swine in Belgium. Since then several H1N1, H1N2, H3N2, and A(H1N1)pdm09 viruses have been detected in commercial and backyard swine as well as in wild boars within Europe. The incidence of IAV in European swine has increased several folds in the past two decades with a relatively high number of IAV positive swine samples (19644/49814). Most of the IAV positive European swine were reported having influenza-like symptoms at the time of sampling. Germany reported the highest number of IAV positive swine in Europe where the pork industry is considered the third largest globally after China and the United States. 

Importantly, the IDV was more recently identified in the European swine, first in Italy in 2015, and later a retrospective study identified IDV infection in swine samples collected in Luxembourg during 2014–2015 which indicated that the circulation of IDV in European swine took place only after 2014. The evidence has suggested the bovine to swine transmission of IDV. This observation is interesting because until recently more emphasis has been given to the avian-swine interaction and the bovine–swine interactions have been neglected from the influenza virus spill over perspective.

The first occurrence of IAV in Asian swine can be traced back to 1969, but the IAV prevalence has increased multi-fold in the recent two decades. The IAV subtypes H1N1, H1N2, H3N2, and A(H1N1)pdm09 have become endemic in swine in several Asian countries. The highly pathogenic avian-origin IAV strains of H5N1 and H7N9 have been reported from Chinese swine while H5N1 has been documented in swine in Viet Nam and Indonesia. The highly pathogenic strains of H5N2 have been reported in South Korean swine. Several LPAIV strains including H4N1, H4N6, H4N8, H6N6, H9N2, and H10N5 have also been documented in Chinese swine. The studies suggested a frequent interaction between wild birds and swine in China which appeared to have transmitted avian-origin IAV strains in the Chinese swine. Occurrence of equine influenza virus H3N8 in Chinese swine further expanded the genetic diversity of swine influenza viruses.

Despite an avian to human transmission of certain avian influenza virus strains including H5N1 virus, only a limited human to human transmission of avian influenza viruses was established in the past [350,351]. With the passage of these avian-origin IAV strains in a mammalian host like swine, a high probability remains of these avian influenza virus strains to adapt and gain the ability of the human to human transmission, if happens, the consequences would be devastating for the public health. 

Australian swine were free from influenza virus until the year 2009 when a New South Wales swine farm reported an influenza-like outbreak in the swine. The zoonotic transmission of the A(H1N1)pdm09 virus was reported to the farm workers and the farm owner. Until now, there have only been four IAV reports in Australian swine which reflects a low prevalence of IAV in Australian swine. New Zealand is yet to officially report the influenza virus prevalence in swine and remains free from the disease. 

A retrospective study identified that the A(H1N1)pdm09 virus was present in Mexican swine as early as 2000, well before the influenza pandemic occurred during March–May 2009. A high genetic diversity of IAV in Mexican swine due to live swine imports from North America and Europe during 1980s laid the foundation of the emergence of zoonotic strain of A(H1N1)pdm09 virus in Mexican swine [33]. The highest number of IAV positive swine in Central America were reported from Mexico followed by Guatemala. Interestingly, a report of the highly pathogenic H5N2 virus in Mexican swine in 2018 further triggers the alarm in the context of a potential novel IAV reassortment. Outbreaks of IAV in the South American swine populations occurred during the last two decades, with the highest prevalence reported in the Brazilian swine. A considerable proportion of cases showed sub-clinical infections with no symptoms which might have made it more difficult to detect the infected swine. 

The reports of IAV active infections or the seroprevalence appeared in the African swine only during last two decades. Until February 2020, IAV have been detected in swine in Cameroon, Nigeria, Egypt, Kenya, Reunion Island, Uganda, Togo, and Ghana. However, South Africa has a considerable swine population [352], but currently there is no published report on the prevalence of active IAV or other influenza virus infections in the South African swine. This might be because of the lack of an active surveillance for the detection of the influenza virus disease in the swine in South Africa.

## 5. Conclusions

The reports of reassortant, double-reassortant and triple-reassortant influenza viruses in Asian, North American and European swine strengthens the concept of swine being the “mixing vessel” in terms of influenza virus reassortment and evolution. The multiple reports of avian-origin IAV strains including highly pathogenic H5N1, H5N2 and H7N9 in swine are alarming given the fact that the avian-origin strains may adapt in swine to facilitate the emergence of a reassortant pandemic strain. The highest number of influenza virus studies in swine population have been reported from the United States (*n* = 40) followed by China (*n* = 39). Also, the United States reported the highest numbers of IAV cases in swine. Due to widespread active surveillance, the United States has significantly brought down the influenza virus disease in swine in the last two decades. Conversely, the IAV disease burden has increased multi-fold in Chinese swine in the last two decades. Additionally, the occurrence of several high- and low-pathogenic avian-origin IAV strains in the Chinese swine population may put the country at greater risk of an influenza pandemic for the future. Given the “mixing vessel” nature of swine physiology, the occurrence of several avian-origin IAV strains and multiple reports of double-reassortant and triple-reassortant IAV subtypes in Chinese swine are alarming because reassortments in swine may facilitate the emergence of a new IAV strain of pandemic potential. 

In the background of the current Corona virus pandemic (COVID-19) which originated in China, the presence of avian-origin IAV strains in Chinese swine must be considered a serious threat for the future and hence must be dealt accordingly. An active nationwide swine surveillance similar to that of North America which as a result, has brought down the current prevalence of influenza virus in the North American swine, should be in place in the rest of the world to safeguard the public health and the economics of the swine farming. A better and active worldwide swine influenza surveillance would be useful for upgrading the current diagnostic protocols and vaccines to prevent future influenza virus outbreaks. 

## Figures and Tables

**Table 1:** Prevalence of Influenza viruses in swine populations worldwide.

Continents	Countries	Influenza A Virus (IAV) Subtypes	Other Influenza Virus Types	References
**Africa**	Cameroon	H1N1, A(H1N1)pdm09	None	[51,52,53]
	Nigeria	H1N1, H3N2, A(H1N1)pdm09, H5N1	None	[52,54,55,56,57,58,59]
	Ghana	H3N2	None	[57]
	Egypt	H5N1, H5N2, H9N2, A(H1N1)pdm09	None	[60,61]
	Kenya	H1N1, H3N2, A(H1N1)pdm09	None	[62,63,64]
	Benin, Cote d’Ivoire	None	None	[65]
	Reunion island	A(H1N1)pdm09	None	[66]
	Togo	A(H1N1)pdm09	None	[67]
	Uganda	IAV	None	[68]
**Asia**	China	H1N1, H1N2, H3N2, A(H1N1)pdm09, H5N1, H9N2, H4N1, H4N6, H5N3, H10N5, H4N8, H6N6, H7N9, H3N8	ICV, IDV	[69,70,71,72,73,74,75,76,77,78,79,80,81,82,83,84,85,86,87,88,89,90,91,92,93,94,95,96,97,98,99,100,101,102,103,104,105,106]
	Bhutan	H1N1, A(H1N1)pdm09	None	[107]
	Cambodia	H1N1, H3N2, A(H1N1)pdm09	None	[108,109]
	Japan	H1N1, H1N2, H3N2, A(H1N1)pdm09	ICV	[110,111,112,113,114,115,116,117,118,119,120,121,122,123,124,125,126]
	South Korea	H1N1, H1N2, H3N2, A(H1N1)pdm09, H7N2, H5N2, H3N1	None	[127,128,129,130,131,132,133,134,135,136,137,138,139,140,141,142,143]
	Thailand	H1N1, H1N2, H3N2, H3N1, A(H1N1)pdm09	None	[144,145,146,147,148,149,150,151,152,153,154,155,156,157,158,159]
	Viet Nam	H1N1, H1N2, H3N2, A(H1N1)pdm09, H5N1	None	[160,161,162,163]
	India	H1N1, H2N2, H3N2, A(H1N1)pdm09	None	[164,165]
	Lebanon	H9N2	None	[166]
	Malaysia	H1N1, H3N2	None	[167]
	Laos	H3N2	None	[168]
	Russia	H1N1	None	[169]
	Taiwan	IAV	IBV	[170,171]
	Indonesia	H5N1	None	[172]
	Sri Lanka	H3N2, A(H1N1)pdm09	None	[173]
	Kazakhstan	H1N1, H3N2	None	[174]
**Australia**	Australia	H1N1, H1N2, H3N2, A(H1N1)pdm09	None	[175,176,177,178]
**Europe**	Belgium	H1N1, H1N2, H3N2	None	[179,180,181]
	Denmark	H1N1, H1N2, H3N2	None	[182,183,184]
	United Kingdom	H1N1, H1N2, H3N2, A(H1N1)pdm09, H1N7	IBV, ICV	[185,186,187,188,189,190,191,192]
	Finland	H1N1, H1N2, H3N2, A(H1N1)pdm09	None	[193,194]
	France	H1N1, H1N2, H3N2, A(H1N1)pdm09	None	[195,196,197,198,199]
	Germany	H1N1, H1N2, H3N2, A(H1N1)pdm09	None	[200,201,202,203]
	Greece	H1N1, H1N2, H3N2, A(H1N1)pdm09	None	[204]
	Italy	H1N1, H1N2, H3N2, A(H1N1)pdm09	IDV	[205,206,207,208,209,210,211,212,213,214,215,216,217]
	Spain	H1N1, H1N2, H3N2	None	[218,219,220,221]
	Netherlands	H1N1, H1N2, H3N2	None	[222]
	Norway	A(H1N1)pdm09	None	[223,224,225,226]
	Poland	H1N1, H1N2, H3N2, A(H1N1)pdm09	None	[227,228,229]
	Czechoslovakia	H3N2	None	[230]
	Hungary	H1N1	None	[231]
	Czech Republic	H1N1, H1N2, H3N2	None	[232]
	Republic of Ireland	H1N1, H1N2, H3N2	None	[232]
	Luxembourg	None	IDV	[46]
	Multiple European Nations	H1N1, H1N2, H3N2	None	[233]
**North America**	Canada	H1N1, H1N2, H3N2, A(H1N1)pdm09, H4N6, H3N3	None	[234,235,236,237,238,239,240,241,242,243,244,245,246,247,248,249,250,251,252,253,254,255,256,257,258]
	USA	H1N1, H1N2, H3N1, H3N2, A(H1N1)pdm09, H4N6, H2N3	IBV, IDV	[259,260,261,262,263,264,265,266,267,268,269,270,271,272,273,274,275,276,277,278,279,280,281,282,283,284,285,286,287,288,289,290,291,292,293,294,295,296,297,298,299,300,301,302,303]
	Mexico	H1N1, H1N2, H3N2, A(H1N1)pdm09, H5N2	None	[304,305,306,307,308,309]
	Guatemala	H3N2, A(H1N1)pdm09	None	[310]
	Cuba	H1N1, A(H1N1)pdm09	None	[311,312]
	Trinidad & Tobago	H3N2, A(H1N1)pdm09	None	[313]
**South America**	Argentina	H1N1, H1N2, H3N2, A(H1N1)pdm09	None	[314,315,316,317,318]
	Brazil	H1N1, H1N2, H3N2, A(H1N1)pdm09	None	[319,320,321,322,323,324,325,326,327,328,329]
	Colombia	A(H1N1)pdm09	None	[330]
	Peru	A(H1N1)pdm09	None	[331]
	Chile	IAV, H1N2	None	[332,333,334]

**Table 2:** An overview of swine sample types and methods used for detection of influenza viruses in swine populations worldwide.

S. No.	Swine Sample Types	Methods Used for Influenza Virus Detection	Virus Types/Subtypes Detected	References
1.	Nasal swab	RNA extraction, real-time RT-PCR, reverse transcription PCR, multiplex RT-PCR, ligation, partial/whole genome Sanger sequencing, Next-generation sequencing, phylogenetic analysis, virus isolation (MDCK cells/Caco-2 cells/HRT18 cells/SPF chicken eggs), transmission electron microscopy, HI assay, NI assay, ultracentrifugation	H1N1, H1N2, H3N2, H4N6, A(H1N1)pdm09, Reassortant H1N1, Reassortant A(H1N1)pdm09, Triple-reassortant H3N2, H1N7, H5N2, IBV, IDV	[5,37,43,45,46,61,74,88,137,157,171,176,188,191,199,207,215,219,220,221,241,243,248,250,263,289,290,301,306,307,326,334]
2.	Tracheal swab	RNA extraction, real-time RT-PCR, reverse transcription PCR, Sanger sequencing, virus isolation (MDCK cells, SPF chicken eggs)	H5N1, IAV, ICV, H1N1, A(H1N1)pdm09,	[38,59,103,321,331]
3.	Nasal wipe	Real-time RT-PCR	H3N2	[289]
4.	Snout wipe	RNA extraction, real-time RT-PCR, virus isolation	IAV	[286]
5.	Oropharyngeal swab	RNA extraction, real-time RT-PCR, Sanger sequencing	IAV, A(H1N1)pdm09	[211,276]
6.	Nasopharyngeal swab	RNA extraction, real-time RT-PCR, virus isolation (SPF chicken eggs), Sanger sequencing	H3N2, A(H1N1)pdm09, IBV, ICV	[38,141,211,277]
7.	Oral fluid	RNA extraction, real-time RT-PCR, virus isolation, Sanger sequencing, Next-generation sequencing (MiSeq)	IAV, IDV, H1N1, H1N2, H3N2, A(H1N1)pdm09	[45,192,227,282,285,287,302,322]
8.	Blood/ serum	IDEXX Ab test, ELISA, HI assay, NI assay, VN assay, MN assay, western blot, virus isolation (MDCK cells)	H1N1, H1N2, H3N2, A(H1N1)pdm09, H5N1, H5N2, H5N3, H9N2, H7N9, H3N1, H4N8, H6N6, H6N2, H7N2, IBV, ICV, IDV	[37,38,46,52,59,70,79,96,97,135,138,153,186,195,200,215,291,306]
9.	Lung/liver/internal organ tissues	RNA extraction, real-time RT-PCR, reverse transcription-PCR, ligation, HI assay, virus isolation (MDCK cells/SPF chicken eggs), Sanger and Next-generation sequencing, hematoxylin-eosin staining, immunohistochemistry, Immunofluorescence	H1N1, H1N2, Reassortant H1N1, H3N2, H2N3, A(H1N1)pdm09, H7N2, IDV	[45,121,127,128,130,201,203,243,245,248,274,314,320,325,327]
10.	Lung homogenate	RNA extraction, real-time RT-PCR, multiplex RT-PCR, single step RT-PCR, virus isolation (MDCK cells/Caco-2 cells/SPF chicken eggs), Sanger sequencing, membrane enzyme immunoassay, HI assay	H1N1, H1N2, H3N2, Reassortant H1N2, A(H1N1)pdm09	[130,213,218,292]
11.	Fecal slurry	RNA extraction, qRT-PCR	IAV	[88]
12.	Rectal swab	Nucleic acid extraction, reverse transcription, metagenomic sequencing	IAV	[300]
